# Substantial but Misunderstood Human Sexual Dimorphism Results Mainly From Sexual Selection on Males and Natural Selection on Females

**DOI:** 10.3389/fpsyg.2022.859931

**Published:** 2022-05-17

**Authors:** William D. Lassek, Steven J. C. Gaulin

**Affiliations:** Department of Anthropology, University of California, Santa Barbara, Santa Barbara, CA, United States

**Keywords:** stature, cephalopelvic disproportion, body fat, muscle mass, disruptive natural selection, neurodevelopmental resources, contest competition

## Abstract

Human sexual dimorphism has been widely misunderstood. A large literature has underestimated the effect of differences in body composition and the role of male contest competition for mates. It is often assumed that sexually dimorphic traits reflect a history of sexual selection, but natural selection frequently builds different phenotypes in males and females. The relatively small sex difference in stature (∼7%) and its decrease during human evolution have been widely presumed to indicate decreased male contest competition for mates. However, females likely increased in stature relative to males in order to successfully deliver large-brained neonates through a bipedally-adapted pelvis. Despite the relatively small differences in stature and body mass (∼16%), there are marked sex differences in body composition. Across multiple samples from groups with different nutrition, males typically have 36% more lean body mass, 65% more muscle mass, and 72% more arm muscle than women, yielding parallel sex differences in strength. These sex differences in muscle and strength are comparable to those seen in primates where sexual selection, arising from aggressive male mating competition, has produced high levels of dimorphism. Body fat percentage shows a reverse pattern, with females having ∼1.6 times more than males and depositing that fat in different body regions than males. We argue that these sex differences in adipose arise mainly from natural selection on women to accumulate neurodevelopmental resources.

## Introduction

In this manuscript we argue that sexual selection acting on males and disruptive natural selection (acting differently on males and females) have made humans a highly sexually dimorphic species, albeit in unique ways. After considering the ways in which these different forms of selection have been understood, we first discuss the modest sex differences in human stature and the critical relationship of paternal and maternal stature to pregnancy outcomes. We then contrast modest sex differences in stature and body mass to the marked sex differences in lean mass, muscle, and strength, and in the percentage and distribution of body fat. We argue that sex differences in muscle and strength are largely the result of sexual selection for male-male competitive traits, whereas females have increased body fat (in place of muscle) due to natural selection for maternal investment capacity in the context of our unusually large brains. Our approach is multifaceted and involves synthetic review of previously published material, some systematic compilations and analysis of data from the literature, and some tests of new hypotheses using new data sets.

Darwin (1859, 1871) distinguished two evolutionary processes of *natural* and *sexual* selection, and much theoretical and empirical work has built on this distinction (for review see Andersson, 1994; Andersson and Iwasa, 1996). Conventional definitions of sexual selection emphasize that it can produce adaptations useful in the mating arena that would not be favored by natural selection (e.g., Darwin, 1871, V1, p. 279). This emphasis is traceable to Darwin’s justification for positing an adaptive force beyond natural selection; if natural selection explains everything, why bother to define an additional process? But he also acknowledged in both 1859 and 1871 that some traits (he used the example of sense organs) could be useful to individuals both in “their ordinary habits of life” and in securing mates, and hence would be favored by both kinds of selection. In other words, adaptive evolution is driven by differentials across an array of fitness components, and that dynamic process has no obligation to be tidy in terms of any particular theoretical framing of natural vs. sexual selection.

Recently, Shuker and Kvarnemo (2021) have attempted to redefine sexual selection as “any selection that arises from fitness differences associated with non-random success in the competition for access to gametes for fertilization” (p. 783). We dislike this formulation because (as explained below) gametes are not always the limiting factor precipitating mating competition, and hence a definition based on them will yield somewhat paradoxical conclusions.

Any definition (or theory) of sexual selection must attend to the fact that mating competition tends to be imbalanced, being more intense in one sex than in the other. That sex is usually, but not always, the male. In anisogamous species, female gametes are larger and thus typically produced in smaller numbers than male gametes. Because it would be expected for males to compete for scarce eggs, but not for females to compete for abundant sperm, sexual dimorphism in gamete size would set the evolutionary stage for disproportionate mating competition among males.

But how would we explain species where female mating competition is more intense; are sperm somehow in short supply? In a ground-breaking paper, Clutton-Brock and Vincent (1991) argued that neither gametes, nor parental investment (*sensu* Trivers, 1972) are the limiting factor driving sexual selection. Instead, they suggested that the sex with the higher potential reproductive rate would inevitably find the “slower” sex in short supply because there would usually be fewer of the slow sex ready to begin a new reproductive venture and they would thus constitute a limited resource for the “faster” sex. Unlike gamete size, where there is a universal sex difference, sex differences in reproductive rate could cut either way, with males or females being the slow sex.

Matching the predictions of Clutton-Brock and Vincent’s (1991) theory, in species where males are the slow sex (e.g., phalaropes), females have evolved to be larger, more brightly colored, and more active in courtship—traits typically associated with mating competition in males. Shuker and Kvarnemo’s (2021) definition would require us to recognize such traits as due to sexual selection when they help males acquire female mates (whose gametes are scarce), but as due to natural selection when they serve the same function for females seeking male mates (because male gametes are not the target of competition). Because that kind of conceptual cleavage seems discordant with Darwin’s original framing of sexual selection as favoring traits useful in mate acquisition, we adopt Andersson’s (1994) widely used definition: “Sexual selection is the differences in reproduction that arise from variation among individuals in traits that affect success in competition over mates and fertilizations” (p. 31).

More generally, adaptive evolution is driven by reproductive differentials between different phenotypes. Those differentials are logically attributable to three fitness components: viability, fecundity, and mating success. Individuals must survive long enough to reproduce and traits that promote such survival are targets of *viability selection*. Among those who survive to reproductive age, some may produce more or better-endowed gametes; some may have better machinery for fostering, protecting or nourishing their young; and some may be more physiologically efficient at converting food into progeny. Such differentials would be targets of *fecundity selection*.

But among those who survive and have efficient, well-formed reproductive machinery, some will get more (or better) mates—because they are more attractive to the opposite sex or because they are better at excluding members of their own sex from mating or a combination of both. These differentials are targets of *sexual selection*. In the terms relevant to this article, viability selection and fecundity selection are both aspects of natural selection, and they stand in contrast to sexual selection. While the conceptual distinction between natural and sexual selection is clear in these terms, the specific fitness differentials shaping any particular dimorphic trait may not be so easily separable because they may not be limited to a single fitness component.

Based on the preceding discussion, it should be clear that disruptive natural selection can produce sexual dimorphism when the ecology of males and females is sufficiently different that viability and/or fecundity selection favor different traits in the two sexes. For example, features of the uterus, placentation, and hormonal support for pregnancy obviously contribute to female reproductive success in ways that would not similarly benefit a male. But these sex differences are the product of natural (more specifically, fecundity) selection, because they benefit female reproduction directly, rather than via any advantage they provide in attracting mates or excluding sexual competitors (although males may subsequently evolve to prefer traits that enhance female reproductive success). Thus, as Darwin (1871) highlighted, the mere existence of a sex difference is not by itself evidence of sexual selection. With these distinctions in mind, we will consider the possible roles of natural and sexual selection in shaping a set of anatomical and physiological differences between women and men.

## Materials and Methods

### Data Relating to Human and Primate Sexual Dimorphism

We used information from seven databases, some previously published and some new. These include data for 260 separate population samples from non-industrial countries with body fat estimated from two skinfolds (Wells, 2012a), a sample of 6,421 persons from 29 countries including 3 in South America, 6 in Africa, and 9 in Europe from 1981 to 2019 (Pontzer et al., 2021), and data on body weights in primates (Smith and Jungers, 1997; Gordon, 2006a,b). From other published data, we also compiled a new dataset of 191 separate population samples that reported values for total mass, fat mass, and lean body mass by sex based on a variety of non-skinfold methods, including underwater weighing, total body water, dual-energy x-ray absorptiometry (DEXA), bioelectrical impedance, MRI, 4-compartment measures, and total body potassium. These 191 samples included 6 foraging populations, 12 horticultural populations, and 23 nations, including some industrial nations (see Supplementary Table 1). In addition, we analyzed data extracted from the 2013 US natality database, the Third National Health and Nutrition Examination Survey (NHANES III, 1988–1994), and from four NHANES surveys for 1999–2006, as detailed below.

### Maternal Stature and Risk of Emergency C-Section

The risk of emergency primary C-section in relation to self-reported maternal stature was determined using logistic regression for 3,550,445 live births, including 1,122,782 primiparas, from birth certificate data from the 2013 US natality database. An emergency C-section was a C-section done in relationship to one or more complications of labor and delivery. Mothers with an elective or repeat C-section were excluded. Control factors included age, parity, race, ethnicity, and birth weight. The relationship between maternal stature and birth weight was also determined with the same controls (except for birth weight).

### Difference in Maternal and Paternal Stature and Risk of Cephalopelvic Disproportion

Data from the NHANES III was used to determine the joint effect of maternal and paternal stature on the risk of cephalopelvic disproportion (CPD). This national U.S. sample included birth certificate data for 10,414 children < 15 years of age, and the presence or absence of a diagnosis of CPD at delivery for 3,190 children. These children were matched to their mothers’ and fathers’ records using the family number, resulting in complete data for 2,918 parent-child triads.

### Sex Differences in Body Composition in the US Based on National Health and Nutrition Examination Survey

Data from NHANES for 1999 to 2000, 2001 to 2002, 2003 to 2004, and 2005 to 2006 were combined into a single data set. Data from dual-energy x-ray absorptiometry (DEXA) were used to assess the amount of fat-free lean tissue, percent body fat, and the ratio of leg fat to trunk fat for 5,273 males and 5,271 females aged 15–49 with a non-obese body mass index of < 30. Sex differences in fat and lean body mass for 10,537 males and 11,536 females aged 18 and older were determined with linear regression controlling for age, race, ethnicity, height, and weight.

Data for 3,878 males and 4,565 females aged 15–39 from NHANES III were used to determine sex differences in waist/hip ratio. In this survey, waist circumference was measured 1 cm above the iliac crest (not at the smallest waist) and hip circumference was measured at its maximum. Comparisons of the two methods of measuring waist circumference in another sample showed that the minimal waist circumference averages 10% less than the “NHANES waist” (Lassek and Gaulin, 2016). Also from NHANES III we determined sex differences in four skinfolds for 16,539 US adults > 17 years old (measured in tenths of millimeters): triceps, subscapular, suprailiac, and thigh.

## Results

### Stature Dimorphism

The various extant primate species differ greatly in body size dimorphism (typically measured as weight—a dimension discussed in more detail below). Most comparative analyses conclude that primate size dimorphism is associated with elevated male contest competition for mating opportunities (Gaulin and Sailer, 1984; Frayer and Wolpoff, 1985; Ely and Kurland, 1989; Plavcan and Schaik, 1997b; Plavcan, 2012a,b). However, there are many other factors—fecundity, life history, feeding niche, predation risk, locomotor substrate, infanticide frequency—that could favor increases or decreases in body size for one or both sexes (Leutenegger and Kelley, 1977; Plavcan and Schaik, 1992; Ford, 1994; Gordon, 2006a,b; Plavcan, 2012a; Cassini, 2020). While undoubtedly important in some cases, these factors tend to be taxon-specific and thus tend not to explain a significant amount of variance in broad comparative studies. As a result, in his comprehensive review Plavcan (2012b) concluded, “to date, male agonistic contest competition is the only factor that has consistently received support from comparative analyses in explaining why males are larger and have larger canine teeth than females in non-human anthropoid primates” (Plavcan, 2012b, p. 52).

A recent meta-analysis across a wide array of taxa is especially relevant because it uses body length dimorphism and is thus comparable to human stature dimorphism (Janicke and Fromonteil, 2021). In their phylogenetically controlled analysis, across 59 species and 95 effect sizes, these authors found a significant positive association between their index of the intensity of pre-copulatory competition for mating partners and the magnitude of length dimorphism. Sexual selection for mating opportunities is a pervasive enough effect that it manifests even when widely diverse taxa—with their unique ecologies and genetic backgrounds—are considered together.

Whereas comparative analyses are an evolutionist’s essential tool for highlighting factors that operate across diverse taxa and circumstances, taxonomically broad relationships do not automatically illuminate particular cases, such as *Homo sapiens*. Forty years ago, J. Patrick Gray compiled world-wide data on human stature dimorphism. Various analyses of that database (Gray and Wolfe, 1980; Wolfe and Gray, 1982a,b; Gaulin and Boster, 1985, 1992, Rogers and Mukherjee, 1992) suggest that the cross-cultural variance in stature dimorphism is both small and uncorrelated with population differences in marriage patterns, subsistence ecology, or mean stature. Sample size, however, is a reasonable predictor: The more males and females were measured in a society, the more its stature dimorphism converged on 1.073 (Gaulin and Boster, 1985).

Wells (2012b) compiled a data set including 96 non-Western populations and also reported a mean stature dimorphism of 1.073. In a larger sample of 137 non-industrialized societies, Wells (2012a) found a mean stature dimorphism of 1.067. In our US sample of 16,343 adults 18–64 from NHANES 1999 to 2006, the ratio is 1.083 (Supplementary Figure 1). The consistency of human stature dimorphism was revealed in a longitudinal context by a study examining Swedish data from the tenth to seventeenth centuries; although mean stature increased as a result of increased nutrition, the magnitude of stature *dimorphism* was unchanged (Gustafsson et al., 2007). In other words, contemporary humans show a very consistent—perhaps genetically constrained (Rogers and Mukherjee, 1992)—level of stature dimorphism, with males significantly taller than females in all known societies.

Importantly for our evolutionary analysis, while the magnitude of stature dimorphism seems to have been relatively stable over recent centuries, it has not been constant over the longer history of the human lineage. Although sexual dimorphism is significantly more difficult to diagnose in the past compared to the present (e.g., Plavcan and Schaik, 1997a; Plavcan, 2012a,b), a consensus persists that sexual dimorphism has decreased over the last 3 million years of hominin evolution (e.g., Richmond and Jungers, 1995; Plavcan and Schaik, 1997a; Lockwood et al., 2007; Gordon et al., 2008). Early, unambiguously bipedal hominins (e.g., members of the genera *Australopithecus* and *Paranthropus*), were apparently more dimorphic in stature (or in the size of their post-cranial skeletons) than are contemporary *Homo sapiens*. A commonly cited benchmark is that these extinct hominins were roughly as dimorphic as extant gorillas (McHenry, 1991a,b; Richmond and Jungers, 1995; Harmon, 2006; Gordon et al., 2008; Plavcan, 2012b,^2018^; Masao et al., 2016; Villmoare et al., 2019)—a species with intense male contest competition for mates.

Reno et al. (2003) disagreed, suggesting that *Australopithecus afarensis* showed a human-like pattern of dimorphism, but their analysis has been methodologically criticized on multiple grounds, and a reanalysis concurs with the more frequent view that pre-*Homo* hominins were significantly more dimorphic than *Homo sapiens* (Plavcan et al., 2005). Furthermore, recent analyses suggest that early members of the genus *Homo* (approximately 1.5 million years ago) exhibited skeletal dimorphism levels intermediate between *Australopithecus* and contemporary humans (Villmoare et al., 2019).

Many anthropologists, aware of the apparently significant role of sexual selection is shaping sexual dimorphism across species (above), may have been too quick to interpret decreasing sex differences in stature during hominin evolution as evidence for a progressively more monogamous human mating system, and a concomitant decrease in male mating competition. We believe this interpretation deserves more scrutiny because, as demonstrated below, there is significant natural selection on women related to obstetric issues.

#### Effects of Male and Female Stature on Human Parturition

What this predominant sexual-selection perspective neglects is that, as suggested by several primatologists (Leutenegger and Kelley, 1977; Plavcan and Schaik, 1992, 1997b; Ford, 1994; Gordon, 2006a,b; Plavcan, 2012a,b; Cassini, 2020), other fitness components beyond mating competition may be relevant to the evolution of male and female size. A relatively uncontroversial but seldom remarked feature of the transition to *Homo*, is that both sexes increased in stature, but females grew disproportionately larger (McHenry and Coffing, 2000; Carretero et al., 2012; Plavcan, 2018). In other words, stature dimorphism decreased, not because males got smaller (as might be expected if the sexually competitive advantages of large size diminished due to monogamy), but because females increased in stature more than males did. This suggests a need to focus on selection pressures acting uniquely on females, which in turn points to selection components relevant to the female role in reproduction.

Birth weight has fitness consequences for altricial human infants. If they can fit through their mother’s birth canal, heavier infants survive better (Lummaa, 2003). Taller women are advantaged in this respect: In the US natality data set, each centimeter of maternal stature increases birth weight by 11.4, 95% CI [11.3, 11.5] grams. For all births, the risk of having a neonate with low birth weight decreased by 2.6%, 95% CI [2.5, 2.6] for each cm of maternal stature. Without attempting to determine the causal pathway, other studies have found higher reproductive success in taller mothers in Gambia (Sear et al., 2004) and Guatemala (Pollet and Nettle, 2008). A comparative study in 42 developing countries found lower child mortality in the offspring of taller mothers (Monden and Smits, 2009).

Such selective differentials would likely have favored increased female stature, but would they have done so more in *Homo* than in *Australopithecus*? A recent meta-analysis across 60 species of mammals found a strong positive effect of infant birth weight on subsequent survival, with a one SD increase in birth weight increasing the odds of offspring survival by 71% (Ronget et al., 2018). Given the generality of the birth-weight advantage, it would likely have favored increased maternal stature in earlier hominin genera as well as in *Homo*, and thus would not explain a stature-dimorphism reduction in *Homo*. There is, however, another positive selection pressure on female stature that would have operated more strongly in later, larger brained hominins.

The “obstetric dilemma” (Washburn, 1960) arises because selection pressures for efficient bipedal locomotion narrowed the human pelvis in a way that constricted the birth canal. Because the head is the largest part of a fetus, a mismatch between fetal head size and pelvic size—cephalopelvic disproportion (CPD)— became increasingly problematic as hominins evolved larger brains. Human infants are notoriously altricial (helpless) compared to those of our great ape cousins, and this trait is proposed to be an evolutionary compromise to the opposing selection pressures arising out of bipedality and delivering large-brained infants: Deliver the infant earlier in development, before its brain is too large (Krogman, 1951; Rosenberg, 1992; Trevathan, 2010).

While there has been some debate about the obstetric-dilemma explanation for human altriciality (Dunsworth et al., 2012; Haeusler et al., 2021), two key facts are undisputed: Human infants are very large relative to their mothers’ birth canals, and this tight fit is relevant to female fitness (Haeusler et al., 2021; Lassek and Gaulin, 2021). For example, as recently as 1990, there were seven countries (with limited access to surgical delivery) where a woman’s lifetime risk of dying in childbirth was between 8 and 14% (WHO, 2015). The risk of CPD is especially high for a woman’s first birth (Lassek and Gaulin, 2021). Most studies attempting to assess this obstetric risk at different time points in the past and across different types of subsistence ecology agree that the hurdle of delivering a human infant has been an enduring selective agent (Dobbie, 1982; Hogberg and Brostrom, 1985; Arriaza et al., 1988; Headland, 1989; Hill et al., 2007; Haeusler et al., 2021).

Because giving birth is so risky in humans, related selective differentials must have had important influences on the evolving female phenotype. Although bipedality was well established more than 4 million years ago, significant brain expansion began only about 2 million years ago, with the emergence of the genus *Homo.* Evidence of birth canal expansion in a 1.5-million-year-old female *Homo erectus* pelvis from the Afar region of East Africa (Simpson et al., 2008) is consistent with the idea that selective differentials related to obstetric challenges have been reshaping female anatomy for many millennia, and some studies suggest that the female pelvic shape of *Australopithecus* already showed some obstetric-related adaptations (Berge et al., 1984; DeSilva, 2011).

Pelvic expansion is one kind of evolutionary adjustment to the obstetric dilemma but there are biomechanical reasons to think that solution will be constrained by its effects on bipedal locomotion. A wider pelvis increases the valgus angle where the distal femur articulates with the tibia, and that increase places additional stress on the anterior cruciate ligament (ACL). In a college sample, women had an 20% larger tibiofemoral angle than men (Nguyen and Shultz, 2007); and when landing from a short drop, women have a 5–6-degree difference from men in the valgus angle at landing and a 12–13-degree difference in maximum valgus (Russell et al., 2006; Vogt, 2017).

As a consequence of this biomechanical sex difference, women experience two to eight times more ACL tears than men when engaged in the same activities (Arendt and Dick, 1995; Arendt et al., 1999; Gwinn et al., 2000; Agel et al., 2005; Alahmad et al., 2020), and the valgus angle seems to be predictive of such injury in females (Hewett et al., 2005; Russell et al., 2006). A torn ACL would likely have been fatal for an ancestral hominin, thus seriously limiting the net fitness advantages of further pelvic expansion. As typically happens when fitness effects are in opposite directions, selection has traded off the risks of CPD and ACL tears, as evidenced by the fact that both are still relatively frequent.

Selection is opportunistic in the sense that any mutation that, in net, augments fitness will be favored. It seems that increased stature was one such evolutionary innovation. Taller females have, on average, larger pelves without increasing their valgus angles (Adadevoh et al., 1989; Imai et al., 2020). Thus, increases in female stature should reduce the birthing problems associated with the obstetric dilemma. If this view is correct, we would expect a strong positive relationship between female stature and successful delivery of a healthy infant.

**FIGURE 1:**
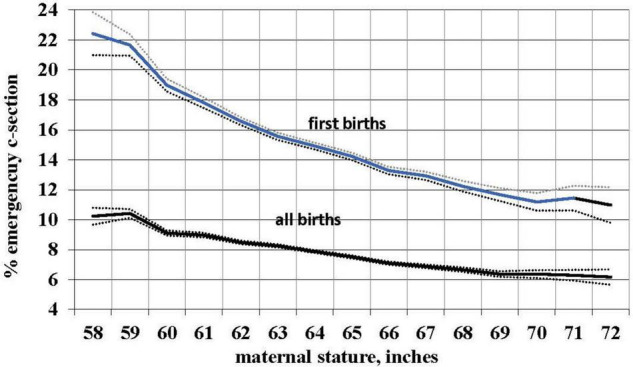
Relationship of mean maternal stature to the risk of an emergency C-section for 3,550,445 total births and 1,122,782 first births with 99% confidence intervals, US, 2013 (without controls).

We used the occurrence of an emergency (i.e., non-elective) primary C-section as a proxy measure for CPD. Using logistic regression with birth certificate data for 3,550,445 live births from the 2013 US natality database, we determined the risk of such C-sections in relation to self-reported maternal stature. As predicted, the risk of a problem delivery decreases with increasing maternal stature. Controlling for maternal age, parity, race, ethnicity, and infant birth weight, the risk of emergency primary C-section declined by 3.2%, 95% CI [3.1, 3.2] with each cm of increase in maternal stature. The uncontrolled risk is shown in Figure 1 and, for first births, declines from more than 22% to about 11% from the shortest to the tallest women. For all births, over the same range of maternal stature the risk declines from more than 10% to about 6%. (First births are of course most relevant because they are the most difficult and because, ancestrally, mothers who did not successfully deliver their first infant never delivered a second.) This result is consistent with smaller studies showing a higher risk of CPD and emergency C-section with shorter maternal stature (Durjardin et al., 1996; Cnattingius et al., 1998; Brabin et al., 2002; Sheiner et al., 2005; Benjamin et al., 2011). Moreover, taller women have a pelvic shape that better accommodates larger-headed fetuses (Fischer and Mitteroecker, 2015).

Because of the contribution of paternal genes to fetal growth rates, the mother and fetus of a union between a taller father and shorter mother should be at increased risk of CPD. Consistent with this prediction, prior studies have shown that larger differences in maternal and paternal stature predict serious complications of labor and delivery. One case-control study (Connolly et al., 2003) found that a one cm increase in maternal stature decreased the risk of CPD (odds ratio 0.91, 95% CI [0.84, 0.98]) while paternal stature was positively related to risk (odds ratio 1.2, 95% CI [1.1, 1.4]), implying that taller fathers and shorter mothers would have the highest risk. This was directly tested in another study (Stulp et al., 2011) which examined the risk of an emergency C-section in relation to the paternal-maternal stature difference and found a significant linear increase in risk as the parental stature difference increased.

For a similar investigation, we constructed a matched dataset of parents and children with birth certificate information for 2,918 mother-father-child triads. Based on logistic regression, for each cm the father was taller than the mother, the risk of CPD increased by 8%, 95% CI [3–13].

The hypothesis that stature dimorphism decreased in order to increase successful delivery was further tested in a cross-national study by Guegan et al. (2000). Since most maternal deaths are due to complications of labor and delivery which are more frequent in shorter mothers, they hypothesized that in populations where there is a high birth rate exposing mothers to more obstetric risk and a high per-birth risk of maternal mortality, there will be a selective pressure for a reduction in stature dimorphism. This hypothesis was supported in an analysis of data from 38 populations. Controlling for other variables, stature dimorphism was lower in populations with higher fertility and maternal mortality.

The results presented above support the idea that as males increased in stature over the last two million years of hominin evolution, female stature increased proportionately more due to natural selection pressures relating to the challenges of delivering larger-brained infants. It was *these combined female and male changes that reduced human stature dimorphism.* Hence, the pervasive belief that the reduction in human stature differences was caused by a parallel reduction in male contest competition is undermined by our analysis of the evolutionarily important relationship between stature and successful parturition. Moreover, other lines of evidence (below) support the idea that sexual selection on males has not abated in the hominin lineage.

### Body Mass Dimorphism

Although sex differences in human body composition confound simple comparisons (see section “Sex Differences in Lean Body Mass, Muscle Mass, and Strength,” below), we review overall body mass because it is the most frequently used measure of sexual dimorphism in studies of other animals. Because of its advantage in aggressive contests, body mass has long been recognized as providing sexually selected benefits for the sex experiencing stronger mating competition (Darwin, 1871; Gaulin and Sailer, 1984; Andersson, 1994; Puts, 2010). Body mass dimorphism is characteristic of non-monogamous primates (Gaulin and Sailer, 1984; Ely and Kurland, 1989; Plavcan, 2012b). Frayer and Wolpoff (1985) cross-tabulated primate body-weight dimorphism by mating system and taxonomic subgroup. In terms of overall body mass, they found that monogamous species ranged from 0% dimorphism among the prosimians to 3.5% among the lesser apes; in contrast, polygamous primates ranged from 10% dimorphism among prosimians to 44% among apes and Old World monkeys.

In contemporary human populations men range from 12 to 25% heavier, on average, than women (Pheasant, 1983; Lindle et al., 1997; Kyle et al., 2005; Xiao et al., 2005). In a notably large sample of 473 Hadza hunter-gatherers, almost equally divided by sex, men averaged 14.9% heavier than women (Sherry and Marlowe, 2007). In samples of 96 non-industrialized populations Wells (2012b) and a sample from 129 nations (Pontzer et al., 2021), men averaged 14.2 and 12% heavier than women. In our dataset of 191 samples, men averaged 21% heavier, and in the US NHANES DEXA database, men were 13.9% heavier. Because women are more likely than men to be obese (Kelly et al., 2008), where obesity is less prevalent, the sex difference in human body mass tends to be larger, as reflected in the larger differences in earlier US samples (Stoudt et al., 1965).

For comparison with related species, males are 10% heavier in gibbons (*Hylobates lar*), 11–32% heavier in common chimpanzees (*Pan troglodytes*), 72–88% heavier in olive baboons (*Papio anubis*), and 66–146% heavier in gorillas (*Gorilla gorilla*) (Smith and Jungers, 1997; Gordon, 2006b). These observed sex differences in overall body mass suggest that *Homo sapiens* falls between monogamous gibbons and promiscuous chimpanzees.

But total body mass provides only a crude picture of the forces shaping these sex differences because, on average, men and women allocate that mass much differently (Plavcan, 2012b). Women have considerably more fat and men have more lean (and muscle) mass (Pond, 1998; Kyle et al., 2005; Wells, 2007, 2012a,b; Lassek and Gaulin, 2009; Hill et al., 2017; Puts et al., in press). Because these sex differences in body composition are present but significantly less pronounced in infants and children and increase dramatically with puberty (Wells, 2007; Kirchengast, 2010; Taylor et al., 2010), this is an ontogenetic sign that they are related to the different reproductive strategies of the two sexes, as explored below.

### Sex Differences in Lean Body Mass, Muscle Mass, and Strength

In contrast to the moderate sex difference in overall body mass, human males have a substantially more (fat-free) lean body mass than females, averaging between 30 and 42% more across the four data sets (Table 1). The overall average of the mean lean body masses was 55.2 kg for males and 40.6 for females, with males having 36% more. For the last two datasets (where they can be calculated), the effect sizes are 1.86 and 2.67. For 96 non-industrial samples (Wells, 2012b), the lean body mass means for males and females were 50.8 ± 1.29 and 38.7 ± 0.77, with males having 29% more with an effect size of 2.34. The human sex difference in lean mass is similar to the 11–32% sex difference in weight for chimpanzees (which have significant contest competition) (above). The sex difference in lean body mass in the NHANES DEXA sample is illustrated in Supplementary Figure 2.

**TABLE 1:** Lean body mass (kg) and percent body fat from four data sets with ratios and effect sizes (*d*) where available.

		Lean body mass, kg					Fat%				
Source	Samples	m	f	m/f	f/m	*d*	m	f	m/f	f/m	*d*
Wells (2012a)	260	51.6	39.7	1.30	0.77		17.0	28.1	0.61	1.66	
Africa	55	47.9	38.3	1.25	0.80		14.9	23.1	0.65	1.55	
Asia	67	45.1	35.0	1.29	0.78		15.5	24.2	0.64	1.56	
Oceania	46	49.9	37.9	1.32	0.76		15.0	24.2	0.62	1.62	
Polynesia	21	60.3	47.6	1.27	0.79		24.6	37.9	0.65	1.54	
S. America	39	50.7	38.0	1.33	0.75		15.6	28.4	0.55	1.81	
Arctic/Subarctic	32	55.5	41.5	1.34	0.75		16.3	30.7	0.53	1.89	
This paper (Supplementary Table 1)	191	58.1	42.1	1.38	0.72		20.1	30.5	0.66	1.60	
Foragers	6	45.9	36.5	1.26	0.80		21.4	32.3	0.63	1.64	
Horticulturalists	12	53.9	38.4	1.41	0.71		13.3	24.6	0.53	1.95	
Non-WEIRD (5)	8	51.4	38.0	1.36	0.74		18.2	31.9	0.57	1.77	
India	8	34.5	26.9	1.28	0.78		18.7	27.2	0.69	1.47	
Japan	8	52.1	37.3	1.40	0.72		18.6	28.7	0.64	1.62	
Canada	8	60.6	43.1	1.41	0.71		19.9	30.2	0.65	1.57	
United States	90	61.9	44.4	1.39	0.72		22.1	32.7	0.68	1.56	
Europe (12)	47	60.6	43.6	1.39	0.72		18.6	28.3	0.66	1.58	
Oceana	8	62.5	45.0	1.39	0.72		21.7	30.8	0.70	1.47	
Pontzer et al. (2021)	129	59.1	44.0	1.34	0.74	1.86	22.8	35.1	0.65	1.55	1.38
16–19	314	57.1	42.5	1.34	0.74	1.96	21.5	32.3	0.67	1.50	1.14
20–25	385	60.3	43.3	1.39	0.72	2.04	19.6	33.6	0.58	1.71	1.56
25–29	467	60.0	43.4	1.38	0.72	2.04	22.3	34.4	0.65	1.54	1.31
30–34	387	59.1	45.2	1.31	0.77	1.68	24.0	36.7	0.65	1.53	1.42
35–39	399	58.9	45.5	1.30	0.77	1.58	26.4	38.4	0.69	1.45	1.48
NHANES 1999–2006*	5,434	56.6	39.8	1.42	0.70	2.67	20.7	32.5	0.64	1.57	2.54
15–19	3,149	53.6	38.6	1.39	0.72	2.32	18.8	31.3	0.60	1.66	2.77
20–25	784	56.3	39.5	1.43	0.70	2.66	19.7	32.2	0.61	1.64	2.78
25–29	541	57.2	40.1	1.43	0.70	2.74	21.4	32.3	0.66	1.50	2.23
30–34	488	57.9	40.4	1.43	0.70	2.75	21.4	33.1	0.65	1.55	2.40
35–39	472	58.2	40.4	1.44	0.69	2.88	22.1	33.5	0.66	1.52	2.50

*BMI < 30, see section “Materials and Methods”.

Lean body mass includes organs, muscle, and bone which, in aggregate, should naturally increase with greater overall body mass. Of these, differences in muscle would be more informative about possible divergent selection pressures on males and females. To assess how much of the lean-mass differences are due to differences in muscle, we compiled 29 samples that measured muscle mass using a variety of methods (Supplementary Table 2) and included new DEXA data from NHANES 1999 to 2006. The mean ratio of male to female muscle mass was 1.65 and the mean effect size (for the 25 studies reporting this statistic) was 2.70.

Perhaps most relevant to aggressive competition in humans is the sex difference in upper-body muscle mass. We have identified 8 studies of sex differences in arm muscle including new data for this paper (Supplementary Table 3). The mean ratio of male to female arm muscle was 1.72 and the mean effect size for 5 studies was 2.91. This is similar in magnitude to the sex difference in lean body mass in gorillas (1.79), the most sexually dimorphic primate (Zihlman and McFarland, 2000). Sex differences in the DEXA sample are illustrated in Supplementary Figure 3. Sex differences in leg muscle mass are somewhat smaller (Supplementary Table 3), but still substantial; data from 14 samples (including new US data) give an average ratio of 1.48 and an average effect size in 11 samples of 2.71.

Contemporary phenotypes suggest that selection has favored larger body size in men than women. Given that background, selection for body-composition differences can be compellingly demonstrated by examining those differences in men and women statistically matched for both height and weight (Table 2). In that controlled analysis, males still have 9.8 kg more lean mass, 2.3 kg more arm muscle, 2.7 kg more leg muscle, and 4.2 kg more lean trunk mass. Thus, human sex differences in body composition are not a mere byproduct of size differences.

**TABLE 2:** Fat and lean body mass differences in 10,537 males and 11,536 females aged 18 and older based on linear regression controlling for age, race, ethnicity, height, and weight, US, NHANES 1999–2006, all *p* < 001.

Measure	Coefficient, 95% CI
Total lean, kg	−9.81 (−9.67, −8.92)
Arm lean, kg	−2.29 (−2.26, −2.31)
Leg lean, kg	−2.74 (−2.68, −2.79)
Trunk lean, kg	−4.20 (−4.13, −4.27)
Fat percent	12.08 (11.94, 12.22)
Total fat, kg	9.81 (9.69, 9.93)
Arm fat, kg	1.31 (1.29, 1.34)
Leg fat, kg	4.92 (4.85, 5.00)
Trunk fat, kg	3.69 (3.61, 3.77)

Muscle mass is a target of selection primarily via the amount of strength it yields. Several relevant meta-analyses of sex differences in strength are available (Sackett and Wilk, 1994; Hough et al., 2001; Courtright et al., 2013) that examine comparable dimensions of strength. All examine muscular tension (exerting force such as pushing, pulling, lifting), muscular power (exerting force quickly), and muscular endurance (exerting force over time), and all measure sex differences not as ratios but as effect sizes (*d*, the number of standard deviations separating males and females), the normal output of meta-analysis. Effect sizes for muscular tension in the three studies were 2.28, 1.86, and 2.13, and for muscular strength in the last two, 1.66 and 1.71.

In addition, Courtright et al. (2013), partitioned their effects by upper-body (arms, chest) vs. lower-body (legs), and found that the sex difference was greater in the upper body (*d* of 1.98 vs. 1.68). All of these effect sizes are very large by conventional standards, in some cases approaching or exceeding two standard deviations. Representative studies from these meta-analyses show that males have twice as much upper body strength as females and 66% more leg strength. When present in other animals, sex differences in physical strength would usually be interpreted as a sexually selected adaptation to aggressive mating competition (Andersson, 1994). Since male muscles are also used in hunting, the possible role of disruptive natural selection related to divergent female and male foraging strategies is also considered in Discussion section “Can Natural Selection Explain Dimorphism in Stature, Mass, and Strength?”.

### Sex Differences in Total Body Fat

Data for the four body-composition datasets (Table 1 and Supplementary Table 1) are in close agreement concerning sex differences in percent body fat: Overall, women have between 1.55 and 1.66 times as much body fat as men. For ages 20–25, the effect size is 1.56 in the sample of Pontzer et al. (2021) and 2.78 in the larger NHANES DEXA sample (illustrated in Supplementary Figure 4). Women not only have substantially more body fat than men, but the average of 30.8 percent fat across the four samples is extraordinarily high compared to mammals generally (Pond, 1978, 1998). Even women undergoing starvation still have significant amounts of body fat. Two groups of women with severe anorexia nervosa (and BMI’s of 16.8 and 16.2) still had 17.2 and 15.2% body fat (Golden et al., 1997).

When adult American males and females are matched for height and weight using regression (see above), females have 12.1% (9.8 kg) more body fat (Table 2), with 1.3 kg more arm fat, 4.9 kg more leg fat, and 3.7 kg more trunk fat (which includes some of the gluteal region). The 12% difference is similar to the samples in Table 1 and gives a female/male ratio of 1.46 for percent fat.

The female body fat percentages in Table 1, persistent over the post-pubertal life cycle, are extraordinarily high compared with most other mammals and primates. Although species differences in body fat are sometimes large (Pond, 1978; Wells, 2006), they are correlated with the unique energy demands of (1) hibernation, (2) long-distance migration, especially in birds, or (3) brief, concentrated reproductive effort (Pond, 1998), none of which apply to humans. Across all mammals for which there are data (including the fatter migratory and hibernating species) mean body fat averages around 7% (Pond and Mattacks, 1985). Thus, men are 2–3 times fatter, and women 3.5–4.5 times fatter (Table 1) than the mammalian average, on a percentage basis, and very much fatter than other species that evolved in tropical savanna environments (Pond, 1978, 1998; Wells, 2006; Puts et al., in press). Possible reasons for women’s larger amount of body fat are the focus of the section “Why Do Women Have so Much Fat?” of the Discussion.

### Sex Differences in Body-Fat Distribution

Not only do men and women differ in the amount of body fat they store, they also differ in where they deposit it. Men deposit most of their fat viscerally whereas women deposit much of theirs peripherally and subcutaneously, especially in the gluteofemoral region (Tichet et al., 1993; Lassek and Gaulin, 2007, 2008; Taylor et al., 2010), giving them a characteristically low waist/hip ratio (WHR). Table 3 shows differences in fat distribution in various national samples. Standard deviations are relatively high because WHR is strongly correlated with BMI, which varies with age. Across the samples, women have 9% lower waist-hip ratios and in the NHANES 1999–2006 sample, a 23% higher ratio of leg fat to trunk fat. There is more than a twofold difference in the depth of the thigh skinfold (Table 3), a measure of subcutaneous gluteofemoral fat, with an effect size of 1.87. Possible reasons for the sex difference in regional fat are the topic of the section “Possible Reasons for Sex Differences in Regional Fat Distribution” of the Discussion.

**TABLE 3:** Sex differences in waist-hip ratio (WHR) and other measures of fat distribution.

Source	Country	n	Ages	Measure	Female	Male	*f/m*	*d*
Wietlisbach et al. (2013)	27 countries	40,480	35–64	WHR	0.81	0.92	0.88	
Tichet et al. (1993)	France	18,393	17–60	WHR	0.79 ± 0.07	0.91 ± 0.07	0.87	1.73
*NHANES III	US	8,080	15–39	WHR	0.84 ± 0.07	0.90 ± 0.07	0.93	0.85
Qiong et al. (2017)	China	2,286	20–29	WHR	0.79 ± 0.06	0.84 ± 0.06	0.94	0.83
Bacopoulou et al. (2015)	Greece	1,610	12–17	WHR	0.73 ± 0.06	0.79 ± 0.06	0.92	1.00
Taylor et al. (2010)	New Zealand	206	20–26	WHR	0.78 ± 0.08	0.85 ± 0.07	0.92	1.00
Fredriks et al. (2005)	Dutch	690	18	WHR	0.75 ± 0.07	0.82 ± 0.06	0.91	1.08
Ahmad et al. (2016)	Malaysia	669	18 +	WHR	0.86 ± 0.08	0.90 ± 0.08	0.96	0,50
Ley et al. (1992)	UK DEXA	234	31–55	Waist fat	12.0 ± 1.9	18.1 ± 2.9	0.66	2.54
				Hip fat	19.4 ± 2.3	18.1 ± 1.8	1.07	0.65
				Ratio	0.62	1.00		
*NHANES 99-06	US DEXA	9,359	15–49	Leg/trunk	1.02 ± 0.31	0.83 ± 0.24	1.23	0.63
*NHANES III	US skinfolds	16,539	>17	Triceps	23.7 ± 8.7	12.6 ± 6.2	1.88	1.48
	mm			Subscap.	22.6 ± 9.5	19.0 ± 7.8	1.19	1.19
				Suprailiac	21.9 ± 10.3	20.9 ± 10.0	1.05	0.09
				Thigh	28.4 ± 9.2	13.3 ± 7.0	2.12	1.87

*New analyses (see section “Materials and Methods”).

## Discussion

As stressed throughout, reliable sex differences are not, by themselves, evidence of sexual selection. As in some other species, natural selection may favor differences between females and males. For example, in raptorial birds the conflicting effects of larger size—increasing fecundity in the sex that lays the eggs, but compromising agility in the sex doing (more of) the hunting—has led to disruptive natural selection such that females are larger than males in many species. This females-larger pattern has evolved independently in the Accipitriformes, Falconiformes, and Strigiformes, groups now known to not be closely related (Schoenjahn et al., 2020).

There are at least two potential sources of divergent natural selection pressures on women and men that might broadly parallel this avian situation: (1) being the sex that gestates and lactates, women might experience certain survival or fertility differentials that are not relevant to men (section “Increase in Female Stature to Accommodate Larger Brained Fetuses”), and (2) a widespread and apparently ancient sexual division of labor might favor different phenotypes in the two sexes, as discussed below.

### Can Natural Selection Explain Dimorphism in Stature, Mass, and Strength?

In addition to sexual selection, natural selection can also produce sex differences in body size, though it seldom favors larger males. For example, natural (i.e., fecundity) selection can favor larger female size, notably in insects and fishes (Perrone, 1978; Thornhill and Alcock, 1983; Andersson, 1994), as realized by Darwin (1871). Likewise, divergent foraging niches can also produce sexually dimorphic body size, perhaps the best examples being raptorial birds where, again, females are consistently larger than males (Newton, 1979; Andersson and Norberg, 1981; Schoenjahn et al., 2020).

A sexual division of labor, with males concentrating on hunting mobile prey and women focusing on immobile plant foods is nearly universal among human foragers (Murdock, 1937; Gurven and Hill, 2009) and possibly primitive in the chimp-human clade given that hunting, while rare, is a nearly exclusive male activity in common chimpanzees (Mitani and Watts, 2001; Mitani et al., 2002). It has been suggested that this ecological sex difference could have generated natural selection for sexual dimorphism in human body size (Kaplan et al., 2000).

Evidence from modern hunter-gatherer groups suggests that male hunting provided a major portion of calories, fat, and protein to their families (and other families of their group through meat sharing) during human evolution (Cordain et al., 2000; Gurven and Hill, 2009). Given that men’s hunting ability correlates positively with their muscle mass (Apicella, 2014) perhaps *natural* selection played a role in shaping both the sexual division of labor and sex differences in muscle mass.

The evidence on this issue is scant and indirect. Wolfe and Gray (1982b) conducted a cross-cultural analyses of stature dimorphism in relation to mode of subsistence. They found that, compared to hunter-gatherers, agriculturalists (whose sexual division of labor is less marked) actually exhibit more stature dimorphism and that, overall, a more equal division of labor was not associated with a reduction in stature dimorphism.

Based on a different cross-cultural sample, Holden and Mace (1999) used a sophisticated analytical strategy that controlled for both cultural and geographic relatedness among their sample populations. In their analyses, foraging populations showed the same levels of stature dimorphism as farming populations. However, dividing their cases differently, they found that cultures where women provide a higher proportion of food than men (regardless of how food is procured) showed less stature dimorphism. They suggested that this was the result of female offspring being better nourished in populations where women are the primary providers. In support of this interpretation they showed that sex biases in juvenile mortality showed a parallel pattern—juvenile males survive better where men contribute more to subsistence and juvenile females survive better when women contribute mere.

Using a genomic approach (Arner et al., 2021), examined allelic differences associated with a number of sexually dimorphic human phenotypes including height and body mass. They found no evidence of post-agriculture changes in the intensity of selection at these loci. In other words, the available evidence indicates that changes in subsistence have not had appreciable effects of human dimorphism, suggesting that human dimorphism is not primarily shaped by selection related to subsistence.

### Dimorphism and Male Mating Success

Sexual selection arising out of competition for mates can cause a wide array of sex differences, but they broadly cluster into two major types: those that increase attractiveness to the opposite sex, and those that increase the ability to exclude same-sex competitors from mating (Andersson, 1994). The reasons why mating competition takes one form or the other are not fully understood (but see Gaulin and Sailer, 1984; Puts, 2010 for some suggestions), but the former is predominant in birds and the latter predominant in mammals.

How are we to assess the relationship between dimorphism and male mating success over the course of human evolution? A meta-analysis of the relationship between “status” (measured variously as physical formidability, hunting ability, material wealth, and political influence) and several male fitness components across 33 non-industrial societies found a consistent positive effect, but a weaker effect than in non-human primates (von Rueden and Jaeggi, 2016) which could be taken to indicate a weakening of male contest competition in extant humans accompanying the spread of monogamous marriage systems. A study of Y-chromosome distribution found evidence of a recent shift from polygyny to monogamy (Dupanloup et al., 2003).

However, contemporary fitness effects may not be relevant to explaining the selective forces that produced the extant pattern of human sex differences. It is a cornerstone of evolutionary explanation that current traits are the result of selection pressures that operated in ancestral populations (Tooby and Cosmides, 1990). Thus, if the present is different from the past, the fitness differentials that produced the trait may no longer obtain. Of possible relevance to reconstructing selective regimes, we can discover no law prohibiting polygamy earlier that the (British) Bigamy Act 1603.

Because we have no direct evidence of the human matting system during the period when human patterns of dimorphism were shaped, it seems reasonable to give more weight to the genetic evidence provided by the Y-chromosome. Genetic studies uniformly find much less genetic diversity in the human Y-chromosome than in human mitochondrial DNA which is passed only through females. This is consistent with a smaller effective male population size and higher variability in male than female reproductive success (Destro-Bisol et al., 2004; Wade and Shulter, 2004; Wilder et al., 2004a,b; Shriver, 2005; Hammer et al., 2008, 2010; Favre and Sornette, 2012; Heyer et al., 2012; Balaresque et al., 2015; Karmin et al., 2015; Poznik et al., 2016). This is also reflected in demographic data showing higher variance in male reproductive success across a range of tribal societies (Betzig, 2012).

Although a sexual division of labor might generate natural selection for hunting ability, there is substantial evidence that dimorphism related to hunting ability has been under *sexual* selection. In Hadza hunter-gatherers, upper-body strength predicts hunting success, prestige, and reproductive success (Apicella, 2014) and there are similar benefits in the Tsimane (Gurven and von Rueden, 2006) and Ache (Hawkes, 1991). Successful hunters achieve elevated reproductive success through a number of pathways (e.g., Kaplan and Hill, 1985; Smith, 2004). Hunting may provide males a costly signaling venue to display traits attractive to females (Bliege-Bird et al., 2001). In other words, sexual division of labor itself may be, at least in part, a consequence of disproportionate male mating competition. The same muscular attributes useful in hunting may also have been sexually selected by providing advantages in aggressive competition among men (or groups of men, see Zeng et al., 2018) for access to mates.

In addition to male muscularity serving a direct role in competing with other males, a number of studies have shown that it is also associated with female judgments of male attractiveness (Horvath, 1981; Dixson et al., 2003; Fan et al., 2005; Geary, 2005; Dixson and Dixson, 2007; Frederick and Haselton, 2007; Honekopp et al., 2007; Dixson et al., 2010b) and with reproductive success in China (Schooling et al., 2011), although men may overestimate how much muscle mass is optimally attractive (Lei and Perrett, 2021). These female preferences would have evolved only if there were preexisting fitness benefits to male muscularity (e.g., in contest competition for mates or in hunting).

Sex differences in body mass, muscle mass, and strength are typical outcomes of sexual selection in mammals generally and primates in particular. Their frequency in related species does not guarantee that they were caused by the same sexual-selection processes in humans. However, our confidence that they were will be increased if we see evidence that male mating competition had produced sexual dimorphism in other traits that are not obviously related to hunting.

We can begin with the observation that males are the “fast” sex (*sensu* Clutton-Brock and Vincent, 1991) in humans, returning to the mate pool more quickly and thus more likely to find mates scarce. Quantitative evidence indicates that more men are excluded from fatherhood than there are women excluded from motherhood, the essential consequence of differential mating competition. For example, there is higher variance in male than female reproductive success across a range of societies and that variance difference increases as populations become more sedentary (Betzig, 2012).

If men evolved to exclude competitors from mating they should have psychological traits designed for that purpose, such as increased aggression, and many kinds of data indicate that men are more physically aggressive than women (Archer, 2009). An indication of how pervasive fighting between young males is in the US can be is found in the results of biennial surveys of American high school students. From 1993 to 2019, an average of 39% of males vs. 23% of females in grades 9–12 in American schools were involved in one or more physical fights in the previous 12 months (NCES, 2021).

A number of studies have found a positive relationship between male reproductive or mating success and aggression (Sadalla et al., 1987; Chagnon, 1988; Connolly et al., 2000; Charles and Egan, 2004; Llaurens et al., 2009) and physical dominance (Puts et al., 2006; Gangestad et al., 2007; Markey and Markey, 2007; Bryan et al., 2011) supporting the idea that human male aggression is the result of sexual selection (Archer, 2009; Georgiev et al., 2013), as it in other mammals (Gómez et al., 2016). Human females are more prone to choose aggressive and combative males as mates when they feel in danger from other males (Snyder et al., 2011).

The more extreme the nature of the violence, the more extreme are the observed sex differences. Because it is less likely to be an effect of differential socialization by sex, the least contaminated assay of evolved sex differences in aggressive tendencies is same-sex homicide outside of warfare (within-society homicides), because they are nowhere condoned for either sex. Nevertheless, a very large sex difference remains. Men are much more likely to kill a man than a woman is to kill a woman; the discrepancy is large and relatively stable across time and across different modes of subsistence, with males accounting for approximately 95% of all same-sex homicides (Daly and Wilson, 1990). The most common “motive,” according to standard federal reporting criteria, is not robbery but “incident of trivial origin”—status competition that went further (perhaps) than the competitors expected (Daly and Wilson, 1988).

Homicide related to mating competition is a frequent occurrence in many hunter-gatherer societies (Kruger and Fitzgerald, 2012; Allen and Jones, 2014), including the “harmless”! Kung, in which most homicides are related to competition for women (Lee, 1993). In the Gebusi, for example, a group with a very high homicide rate of 7 per 1,000, the professed cause was to punish sorcery but the actual cause was to increase male control of marriageable women (Knauft, 1987). Across cultures, time, and species, lethal male-male aggression is strongly related to mating competition (Kruger and Fitzgerald, 2012).

In that context it is relevant that men’s (but not women’s) proneness to anger and history of physical aggression are both correlated with their own physical strength, suggesting psychological mechanisms for the strategic deployment of aggression against conspecifics (Sell et al., 2009); “don’t start it unless you can win it.”

Of course, in addition to competition between individual men within groups, there is also substantial evidence for persistent violent competition between groups in the form of warfare or raiding, which is the most common form of killing seen in chimpanzees (Bowles, 2009; Puts, 2010; Allen and Jones, 2014; Puts et al., 2015; Puts, 2016; Hill et al., 2017; Mann, 2018). Competition between patrilineal groups has been suggested as the cause of a post-Neolithic Y-chromosome bottleneck (Zeng et al., 2018).

When men attack each other (in the absence of weapons) fists slamming into faces is a common pattern. Sexual selection may have been at work in this domain as well. The particular facial bones most frequently fractured in such altercations evolved greater robusticity such that they have been for several million years, and continue to be, the most sexually dimorphic elements of the human skull (Carrier and Morgan, 2015), suggesting that such conflict was predominantly a male-male affair.

If faces are targets of attack in male contest competition, selection may have produced beards as adaptive protection. Beards are a notably derived (e.g., compared to chimpanzees, where the area around the mouth is one of the least hairy regions), and highly dimorphic feature of human anatomy (Darwin, 1871). Facial hair is morphologically distinct from scalp hair in ways that may allow it to deflect and/or absorb blows to the face (Beseris et al., 2020). Men perceive potential competitors with full beards as more dominant (Puts, 2010; Dixson and Vasey, 2012; Dixson et al., 2017a). As we would expect if it plays a role in male contest competition, beardedness is more prevalent in countries with male-biased sex ratios (Dixson et al., 2019), under crowded conditions (Dixson et al., 2017b), and where economic inequality is high (Dixson and Lee, 2020; Pazhoohi and Kingstone, 2020). Compared to clean-shaven faces, beards enhance judgments of male facial masculinity, dominance, and aggressiveness, irrespective of underlying facial structure (Sherlock et al., 2017; Mefodeva et al., 2020). Likewise, beards enhance the speed and accuracy of detecting an angry facial expression (Craig et al., 2019; Dixson et al., 2021).

If beards are a sexual display, which sex are they displaying to? Several studies that compared the effects of beards on dominance or aggressiveness judgments by men with their effects on attractiveness judgments by women found the male-male effects significantly stronger (Puts, 2010; Dixson and Vasey, 2012; Dixson et al., 2017a). Still, there is some evidence for female preferences, notably that beardedness is more common where pathogen stress is high (Dixson and Lee, 2020; Pazhoohi and Kingstone, 2020), suggesting female choice for good-genes benefits.

Other sexually dimorphic features are even less plausibly related to an adaptive specialization for hunting but apparently related to aggressive male-male interactions. The one-octave sex difference in habitual voice pitch is an example (Puts et al., 2006). Lower voice pitch is much more strongly related to male dominance perceptions than to female attractiveness perceptions (Puts et al., 2007; Hodges-Simeon et al., 2010; Rosenfield et al., 2020), and situational lowering of male voice pitch elevates listeners’ perceptions of the speaker’s aggressive intent (Zhang et al., 2021).

The strong sexual dimorphism in both voice pitch and facial hair results in what animal behaviorists would call male sexual ornaments. Comparative research suggests that men are roughly as ornamented as males in polygynous primate species with large, fluid social groups (Dixson et al., 2005; Grueter et al., 2015). Beards and low-pitched voices are especially relevant to our analysis because an array of experimental studies links them to aggressive male-male competition in a way that they cannot be plausibly linked to the alternative hypothesis of selection for hunting abilities.

Lastly, ontogeny also provides relevant evidence about the evolution of human sexual dimorphism. The sex differences that are the focus of our article—stature, body mass, body fat and lean mass, fat distribution, muscularity and strength—all emerge or are significantly amplified around sexual maturity. This timing suggests they are related to reproduction rather than to sex-specific ecological adaptations.

### Increase in Female Stature to Accommodate Larger Brained Fetuses

Gestation and lactation are demanding mechanically, energetically, and in terms of supplying particular nutrients to the fetus (e.g., calcium for the skeleton, fat for the brain). Because males do not face these demands it would be surprising if disruptive natural selection had not caused female and male phenotypes to diverge with respect to these demands. We have suggested two such adaptations.

One is the increase in female stature relative to males. With the transition to larger-brained *Homo* from earlier *Australopithecus*, sexual dimorphism in stature decreased. This decrease is widely regarded as indicating a shift to a more monogamous mating system with a concomitant decrease in male mating competition (a sexual-selection explanation). But this explanation ignores potentially relevant facts: The decrease in stature dimorphism was accomplished via size increases in both sexes, with the female size increase being relatively greater than the male.

We interpret these facts as indicating some novel selection pressure on females, and specifically highlight the difficulty of delivering a large-brained infant.

Our analyses of contemporary data show that taller women, and women closer in stature to their mates, are less likely to experience an emergency Caesarian section (a proxy for the kinds of birthing difficulties that would have reduced female fitness during hominin evolution). To the extent that our data are relevant, the current level of human stature dimorphism seems: (1) to have been significantly shaped by viability and fecundity selection on women and, (2) consequently, underestimates the intensity of sexual selection on males.

### Why Do Women Have so Much Fat?

The second adaptation in human females likely brought about by natural selection is the increase in the percentage of body fat. Currently, there is no scientific consensus about why women (and to a lesser extent men) have such high levels of body fat. Fat is heavy and, unlike muscle, does not contribute to its transport costs. For this reason, no animal should store more fat than it needs.

One suggestion is that hairlessness increased our need for insulation against hypothermia (Kushlan, 1980) although, even if correct, this idea would not explain the sex difference. Pond (1992) has presented comparative mammalian evidence from the *Carnivora*, an order with species in a wide array of habitats from tropical to polar, and found no support for the idea that fat deposits evolved primarily for insulation. Moreover, hominins were a tropical lineage throughout most of their history with relatively little need for insulation. If insulation were the main purpose of human fat we should have very little, or there should be large population differences in body fat—correlated with latitude—that evolved as our ancestors began moving out of Africa.

Wells (2012a) shows a very weak positive correlation between peripheral body fat (as assayed by triceps skinfold) and latitude, but only when he omits the Polynesian populations from his sample. Regardless, tropical African hunter-gatherers such as the Hadza at 10.6–13.5% body fat for men and 19.0–20.9% body fat for women (Sherry and Marlowe, 2007; Pontzer et al., 2012) have far too much adipose to support the insulation hypothesis for high levels of human fat deposition.

The obesity epidemic, while real, is also not the explanation, because Wells’s (2012a) sample of non-industrial populations (Table 1) and our sample of foragers and horticulturalists (Table 1 and Supplementary Table 1) show that high levels of body fat are human universals.

Because only women gestate and lactate, this sex difference is widely assumed to have favored their disproportionate fat deposits (e.g., Frisch, 1984; Power and Schulkin, 2008; Kirchengast, 2010). But if this were the correct explanation, all mammals should exhibit similar sex differences in body fat. In contradiction to this expectation, significant sex differences in total fat deposition are not the norm in mammals (Pond, 1978; Pond and Mattacks, 1985) nor in primates, and sometimes are skewed in the opposite direction with males being fatter (*Macaca fasicularis*: Pond and Mattacks, 1987; *Papio anubis*: Eley et al., 1989; *Papio cynocephalus*: Altmann et al., 1993; *Macaca mulata*: Colman et al., 1998; *Callithrix jacchus*: Power et al., 2001; *Macaca fuscata*: Hamada et al., 2003; *Propithecus verreauxi*: Lewis and Kappeler, 2005; Raman et al., 2005).

Could the higher fat levels in human females be due to their needing more energy during pregnancy and lactation than other primates? This seems unlikely. Studies show that the energy costs of pregnancy and lactation in relation to maternal weight in humans are similar to other primates and apes, and primate females typically deal with the energy costs of pregnancy and lactation by increasing food intake (Dufour and Slather, 2002).

Although primates generally have longer gestations than other mammals, thus decreasing their daily energy requirement, the length of gestation in humans in relation to the mother’s weight is close the primate regression line (Dufour and Slather, 2002) and daily maternal energy investment is also on the regression line for other apes (Ulijaszek, 2002). Human lactation costs are also similar to other primates. The lactation period for human females (based on the !Kung) is below the regression line for primates and apes (Dufour and Slather, 2002). The relatively dilute concentration of nutrients in human milk is similar to other primates (Dufour and Slather, 2002) and the calories per gram are lower than in baboons and other monkeys (Oftedal, 1984). Women’s cost of lactation in relation to weight is much lower than in many other mammals and similar to baboons (Prentice, 1988). In other words, species differences in the *energetic* costs of reproduction would not seem to demand greater stored resources in women than in our primate relatives.

In species with well-defined breeding periods, males may seasonally accumulate additional fat to facilitate aggressive competition for mates (Boinski, 1987; Bercovitch, 1992) or females may do so to support their maternal investment (Hamada et al., 2003). But in her comprehensive review of fatty tissues in wild vertebrates, Pond (1978) remarked that the *perpetually* large fat deposits in human females are very unusual.

The exceptional nature of women’s high fat storage is further underscored by a comparative analysis across 87 mammal taxa which showed that, in species with alloparental care—whether by the father or others—females store less fat (Heldstab et al., 2017) presumably because, by providing some resources, fathers and allomothers reduce the demands on mothers. It has been estimated that alloparenting decreases the lifetime reproductive effort of human females by 14–29% compared with other mammals (Bogin et al., 2014). Thus, to be as large they are, to manifest relatively early in development, and to be so permanent in a species with significant alloparental care, women’s fat deposits must have been shaped by both strong and relatively unique selection pressures.

Perhaps women’s higher percentage of fat is explained by another unique human trait: our large brains. All female mammals must provision the development of their fetus and infant, but none must build as large a brain in proportion to their own body size. Brains not only consume large amounts of energy from glucose, but they require significant amounts of quite specific fats—notably, long-chain omega-6 and omega-3 polyunsaturated fatty acids—as major building blocks.

On a dry-weight basis, the human brain is about 60% fat (Bradbury, 2011), and two rare long-chain fatty acids, the omega-3 docosahexaenoic acid (DHA) and omega-6 arachidonic acid, each constitute about 10% of brain fatty acids (Makrides et al., 1994), with DHA playing the most critical role in brain development and function (Lauritzen et al., 2016). These essential fatty acids cannot be synthesized by humans and thus must come from the diet and be stored until needed.

The percentage of DHA in stored fatty acids reflects the percentage in the long-term diet and is seldom more than 0.4% of total adipose (Knutsen et al., 2003; Luxwolda et al., 2014). However, when there is less DHA in the diet, the concentration of DHA in stored adipose is consequently lower, and the only way to store more DHA is to increase the total amount of adipose (and hence BMI). Women with less DHA in the diet thus tend to have more fat: Female BMI is inversely related to the amount of DHA in the blood (Sands et al., 2005). Contemporary studies indicate that DHA is preferentially stored in gluteofemoral fat and then mobilized during pregnancy and lactation (see “Possible Reasons for Sex Differences in Regional Fat Distribution” below).

This neuro-developmental perspective potentially explains why human mothers need more fat than mothers of other mammalian species (Cunnane and Crawford, 2003; Lassek and Gaulin, 2006, 2007, 2008; Wells, 2006) and also explains why human neonates have so much body fat (Cunnane and Crawford, 2003; Correia et al., 2004). Because the amount of various fatty acids that can be stored is proportionate to their occurrence in the diet, and because critical omega-3 fatty acids are dietarily scarce, fat stores must be large to contain significant amounts of this critical brain-building fat (Lassek and Gaulin, 2006).

This perspective, if correct, has the advantage of uniting under one explanatory umbrella three highly derived human states: exceptionally large brains, higher levels of body fat than any non-hibernating, non-migrating mammal, and greater sexual dimorphism in body fat than any other mammal. Other hypotheses, such a need for protection against hypothermia, do not fit the comparative data and do not explain the large sex difference. From our perspective, sex differences in the percentage of body fat would be explained by disruptive natural selection favoring larger fat stores in women.

This view contrasts to some degree with the prevailing literature, where women’s fat stores are often viewed as the outcome of sexual selection acting via male choice. Given the extensive evidence concerning male mating preferences for female body shape, there is no doubt that such selection has occurred, but two issues require attention. First, the male preference should not have evolved unless it was targeting existing viability or fecundity differences among females (in other words, such preferences evolved to track what natural selection on females was already favoring). Second, as many studies make clear, the relevant target of male choice is female body-fat *distribution*, as discussed below.

### Possible Reasons for Sex Differences in Regional Fat Distribution

The evolutionary reasons for the sex difference in adipose storage are not fully understood. Mechanically, adding weight in the form of body fat to a limb increases the force required to move it, without increasing strength, so peripheral fat must compromise the agility and power of the arms and legs. That mechanical effect could *dis*favor peripheral fat deposits in the sex competing more intensely for mates—males, as we have argued in the human case. But why would peripheral fat be favored in women? And why should it be localized to a few depots, rather than deposited as a smooth sheath beneath the skin of the whole body?

Various answers have been offered to one or both questions. Positioned near the center of mass of the body, the gluteofemoral depot may stabilize locomotion and provide a counter-balance to the frontal mass of a developing fetus (Pawlowski, 2001; Pawlowski and Grabarczyk, 2003). Another suggestion is that women may shunt fat from visceral to subcutaneous depots to increase the available space for gestation and to reduce intra-abdominal pressure on the fetus (Abrahim, 2021).

Another line of thinking connects to the neurodevelopmental explanation for women’s high levels of body fat (section “Why do Women Have so Much Fat?” above). Relative to other depots, the gluteofemoral depot is protected against use for ordinary energy needs but is then systematically drawn down during late pregnancy and lactation (Rebuffe-Scrive et al., 1985; Rebuffe-Scrive, 1987; Lassek and Gaulin, 2006)—the key period of fetal and infant brain growth.

During lactation most of the long-chain fatty acids in maternal milk come from stored fat rather than from the mother’s current diet (Lassek and Gaulin, 2006), and the gluteofemoral depot seems to be the primary source of the omega-3 and omega-6 fats that are essential for fetal and infant brain development (Lassek and Gaulin, 2007). Women who have adequate food supplies eat substantially less than they need to calorically support lactation and instead mobilize stored fat. Is that to provide critical *materials* stored in that fat?

**FIGURE 2:**
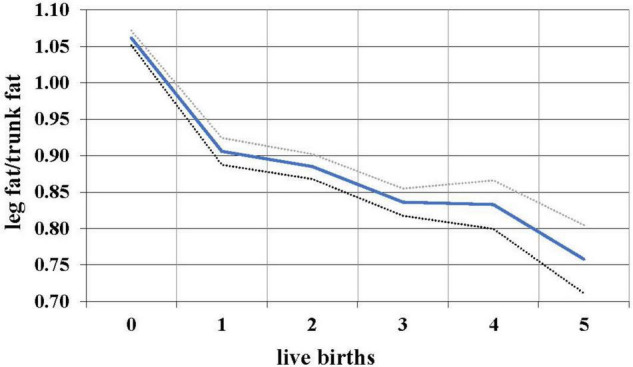
Relationship between live births and leg/trunk fat ratio in 7,753 women 12–49, with 95% confidence intervals, NHANES 1999–2006.

During the female reproductive lifespan, there is a decrease in the relative amount of gluteofemoral fat with parity (Lassek and Gaulin, 2006). Based on data from NHANES 1999 to 2006, the ratio of leg fat to trunk fat (which is highly correlated with the waist-hip ratio, but measures actual fat) drops with each successive birth (Figure 2), suggesting that gluteofemoral resources are differentially consumed in reproduction. Women with a higher thigh/waist ratio have higher levels of DHA, the omega-3 fat found at high levels in the brain (Lassek and Gaulin, 2019). These polyunsaturated fats—especially the omega-3s—are relatively unstable and possibly best stored in cooler subcutaneous rather than warmer visceral locations.

All of the effects on body fat mentioned thus far would result from natural selection, through the more efficient locomotion of the woman or through the production of infants with better-provisioned brains. However, once such selective differentials existed, sexual selection might begin to operate via evolved male preferences for women whose subcutaneous fat deposits indicated superior brain-building potential (Lassek and Gaulin, 2008). A similar argument was advanced by Cant (1981), noting that concentrated fat depots (e.g., buttocks and breasts) more effectively advertised a woman’s stored maternal resources, but did not explicitly identify neurodevelopmental resources as limiting. One study (Szalay and Costello, 1991) attributes all sexually dimorphic fat to sexual selection, but that explanation is incomplete; some pre-existing adipose-related fitness gradient in women is a precondition for the evolution of male preferences that track the gradient.

This logical requirement suggests there was prior natural selection on females for discrete fat depots, perhaps sorted by type of fatty acid and/or intended usage—with male preferences simply evolving to differentially attend to the depots most relevant to female mate quality. Currently, the gluteofemoral depot is the primary source of brain-building fats (above). That fact is consonant with the extensive psychological literature showing that men selectively attend to women’s waist/hip ratios in evaluating female attractiveness (e.g., Singh, 1993; Sugiyama, 2005; Singh and Randall, 2007; Dixson et al., 2010a,b, c, 2011; Brooks et al., 2015), with some researchers arguing that preference data suggest the waist/hip ratio is a supernormal stimulus (Marković, 2017). Several reviews have evaluated and rejected the idea that waist/hip ratios broadly track health and fertility (Lassek and Gaulin, 2018a,b; Bovet, 2019), thus suggesting it provides more specific information content.

## Summary

Over the last two million years of hominin evolution, both sexes increased in body size, but females increased proportionately more. These combined changes produced relatively low levels of stature dimorphism in contemporary humans, a fact that is widely interpreted as indicating a reduction in the intensity of male contest competition for mates over the same time period. But that interpretation fails to recognize the substantial sex differences in human body composition. Men’s much greater lean body mass, muscle mass, and muscular strength (with effect sizes of 2.7–2.9) indicate a degree of dimorphism comparable to other primate species with intense male mating competition. Based on current obstetric evidence, females likely increased in stature relative to males in order to accommodate increasingly large-brained neonates. In parallel, women added body fat (rather than muscle) to provide more of the long-chain fatty acids that are critical for fetal and infant neurodevelopment.

Summarizing these ideas in the light of the two forces of natural and sexual selection: (1) sexual dimorphism in stature, fat mass, and fat distribution have been significantly shaped by disruptive natural-selection regimes operating on females and males, with some likely overlay of subsequent sexual selection acting via mate choice in the case of fat distribution; (2) sexual dimorphism in lean mass, muscle mass, and strength are largely due to sexual selection arising from a long history of aggressive male mating competition, with the some possible influence of divergent natural selection due to sex differences in foraging ecology; and (3) a large literature seems to have overemphasized the role of mate choice, and underestimated the role of male contest competition for mates, in shaping human sex differences.

## Data Availability Statement

Data analyzed from the National Health and Nutrition Examination Survey (NHANES), including the NHANES III and the NHANES 1999–2000, 2001–2002, 2003–2004, and 2005–2006, are available at https://wwwn.cdc.gov/nchs/nhanes/default.aspx. US natality data for 2013 are available from the Period-Linked Birth-Infant Death File for 2013 at https://www.cdc.gov/nchs/data_access/vitalstatsonline.htm.

## Author Contributions

WL compiled the various databases and performed the statistical analyses. SG wrote the first draft of the article. WL and SG jointly developed the ideas presented and edited the article to its final form. Both authors contributed to the article and approved the submitted version.

## Author Disclaimer

Views expressed in this article do not necessarily agree with those of the commentators.

## Conflict of Interest

The authors declare that the research was conducted in the absence of any commercial or financial relationships that could be construed as a potential conflict of interest.

## Publisher’s Note

All claims expressed in this article are solely those of the authors and do not necessarily represent those of their affiliated organizations, or those of the publisher, the editors and the reviewers. Any product that may be evaluated in this article, or claim that may be made by its manufacturer, is not guaranteed or endorsed by the publisher.
