# Supplementary material for: Substantial but Misunderstood Human Sexual Dimorphism Results Mainly From Sexual Selection on Males and Natural Selection on Females
Source: Front Psychol. 2022 May 17;13:859931. doi: 10.3389/fpsyg.2022.859931 (PMC9156798; doi:10.3389/fpsyg.2022.859931)
Supplement: Supplementary file 1 [file Data_Sheet_1.pdf]

SUPPLEMENTARY MATERIAL: Lassek & Gaulin, 2022

Figure S1. Frequency percent distribution of stature in 7,971males and 8,964 females aged 18-64, NHANES 1999-2006.

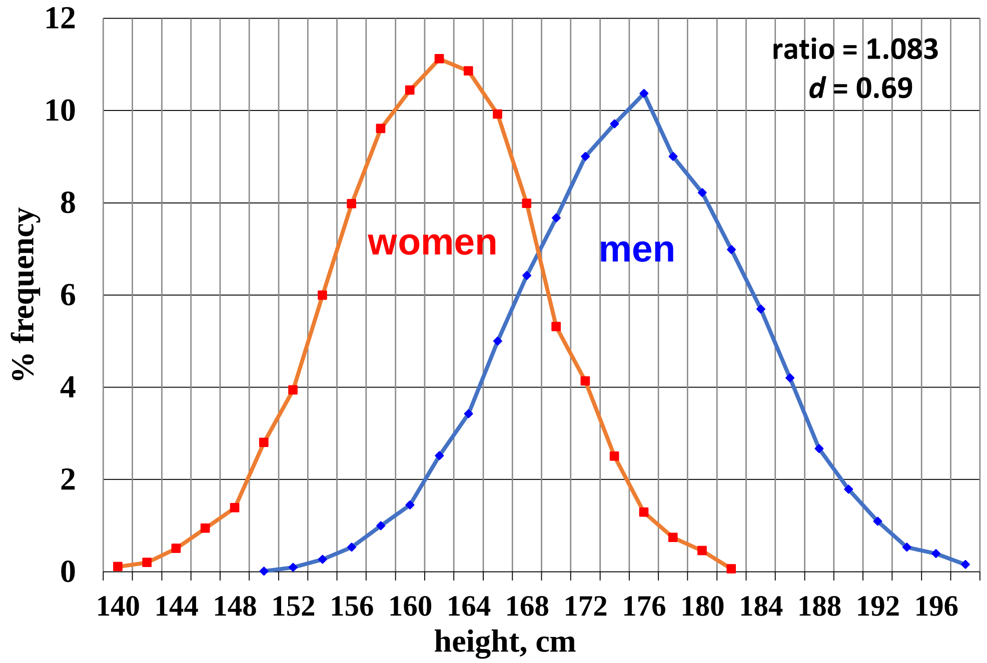

Figure S2. Frequency percent distribution of total lean tissue in 2106 males and 1696 females aged 16-29 with a BMI<25, NHANES 1999-2006.

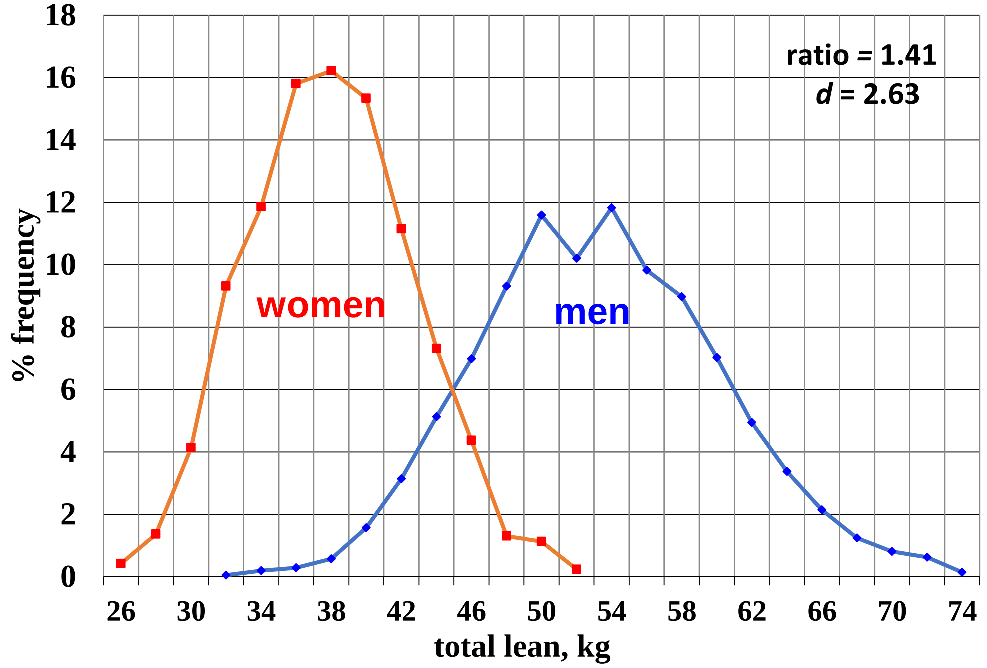

Figure S3. Frequency percent distribution of arm lean weight in 2106 males and 1669 females aged 16-29 with a BMI<25, NHANES 1999-2006.

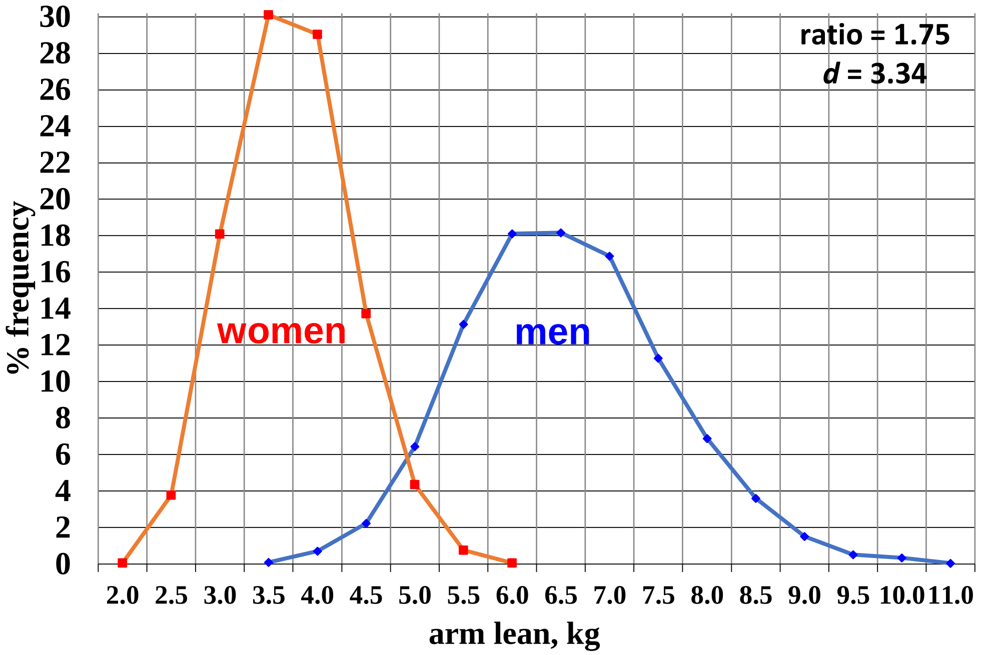

Figure S4. Frequency percent distribution of percent body fat in 2106 males 1696 females aged 16-29 with a BMI<25, NHANES 1999-2006.

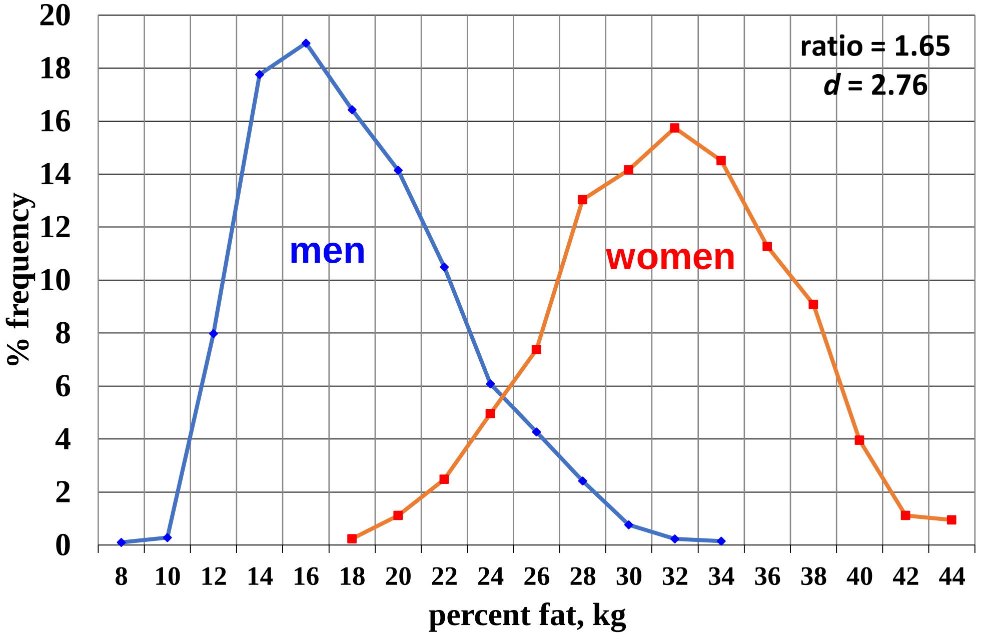

Table S1 Fat free (lean) mass (kg) and percent fat in selected populations

			fat free mass				% fat			
**reference**	**method**	**pop**	**m**	**f**	**m/f**	**f/m**	**m**	**f**	**m/f**	**f/m**
Bribiescas 01	bia	Ache	46.2	36.8	1.26	0.80	17.9	33.3	0.54	1.86
Sherry 07	bia	Hadza	47.6	37.5	1.27	0.79	10.6	19.0	0.56	1.79
Pontzer 12	uww	Hadza	44.1	37.7	1.17	0.85	13.5	20.9	0.65	1.55
Picón-Reátegui 79	deuter	Ainu	48.1	37.2	1.30	0.77	13.6	28.0	0.49	2.06
Kaur 18	bia	Bhil India	41.1	34.3	1.20	0.83	21.3	28.1	0.76	1.32
Sarma 20	sf	BaYaka	48.3	35.7	1.35	0.74	51.7	64.3	0.80	1.24
Norgan 82	uww	Kaul	51.8	39.6	1.31	0.76	8.6	20.5	0.42	2.37
Norgan 82	uww	Kaul	50.8	38.5	1.32	0.76	9.3	21.1	0.44	2.27
Norgan 82	uww	Kaul	49.6	35.8	1.39	0.72	10.3	22.7	0.45	2.20
Norgan 82	uww	Lufa	52.8	39.3	1.34	0.74	9.9	21.9	0.45	2.21
Norgan 82	uww	Lufa	52.3	39.6	1.32	0.76	9.8	22.4	0.44	2.27
Norgan 82	uww	Lufa	53.7	37.2	1.44	0.69	10.1	18.4	0.55	1.83
Christopher 19	uww	Shua	60.7	41.7	1.46	0.69	20.6	28.6	0.72	1.39
Bethancourt 19	bia	Tsimane	51.5	39.4	1.31	0.76	17.7	26.8	0.66	1.51
Gurven 16	bia	Tsiman	52.3	41.8	1.25	0.80	16.0	26.4	0.61	1.65
Snodgrass 05	bia/sf	Yakut	52.5	37.9	1.39	0.72	21.2	37.3	0.57	1.76
Neder 01	dexa	Brazil	57.7	43.8	1.32	0.76	21.8	34.0	0.64	1.56
Liu 95	bia/sf	Chinese	53.4	38.2	1.40	0.72	13.0	27.8	0.47	2.13
Werkman 00	4c	Singapore	64.6	46.1	1.40	0.71	15.9	29.1	0.55	1.83
Werkman 00	4c	Singapore	51.8	36.5	1.42	0.70	19.8	29.9	0.66	1.51
Luke 02	tbw	Nigeria	50.6	39.9	1.27	0.79	15.8	27.0	0.59	1.71
Pongchaiyakul 05	dexa	Thai	47.0	35.2	1.34	0.75	19.7	37.9	0.52	1.93
Pongchaiyakul 05	dexa	Thai	46.8	34.7	1.35	0.74	20.3	36.9	0.55	1.82
Wang 96	bia	China	52.5	39.7	1.32	0.76	19.7	32.7	0.60	1.66
Kuriyan 98	hd	India	43.0	34.6	1.24	0.81	12.6	21.3	0.59	1.69
Kanade 01	bia	India	47.2	35.8	1.32	0.76	21.5	31.0	0.69	1.45
Kshatriya 21	bia	India	32.5	24.9	1.30	0.77	18.4	27.3	0.68	1.48
Kshatriya 21	bia	India	33.2	25.4	1.31	0.77	17.3	25.4	0.68	1.47
Kshatriya 21	bia	India	32.1	26.0	1.23	0.81	17.3	25.1	0.69	1.45
Kshatriya 21	bia	India	31.9	25.1	1.27	0.79	18.8	27.3	0.69	1.45
Kshatriya 21	bia	India	31.8	25.4	1.25	0.80	18.7	27.7	0.68	1.48
Kshatriya 21	bia	India	32.5	25.6	1.27	0.79	15.5	24.3	0.64	1.57
Kshatriya 21	bia	India	31.6	23.8	1.33	0.75	22.1	31.4	0.70	1.42
Kshatriya 21	bia	India	31.7	24.3	1.30	0.77	21.6	30.1	0.72	1.39
Kshatriya 21	bia	India	31.9	24.9	1.28	0.78	22.2	28.2	0.79	1.27
Heitman 91	bia	Denmark					20.6	28.6	0.72	1.39
Schutz 02	bia	Swiss	59.1	42.4	1.39	0.72	19.8	28.4	0.70	1.43
Schutz 02	bia	Swiss	54.5	39.3	1.39	0.72	19.6	27.6	0.71	1.41
Rico 93	dexa	Spain	48.1	33.8	1.42	0.70	12.0	19.3	0.96	1.61
Boot 97	dexa	Dutch	59.1	42.9	1.38	0.73	8.2	26.9	0.31	3.27
Macmillan 65	uww	Scotch	61.1	43.1	1.42	0.71	10.8	25.9	0.42	2.40
Garcia 05	dexa	Germ	65.1	46.5	1.40	0.71	23.0	39.9	0.58	1.74
Brodie 92	bia	UK	59.7	49.4	1.21	0.83	16.7	23.2	0.72	1.38
Rico 92	dexa	Spain	49.2	34.9	1.41	0.71	35.0	36.7	0.95	1.05

			fat free mass				% fat			
**reference**	**method**	**pop**	**m**	**f**	**m/f**	**f/m**	**m**	**f**	**m/f**	**f/m**
Pietrobelli,	dexa	Spain	50.3	37.1	1.36	0.74	17.5	32.5	0.54	1.86
Stewart 93	dexa	UK	59.9	40.8	1.47	0.68	18.2	33.2	0.55	1.82
Han 96	uww	Scot	59.6	42.6	1.40	0.71	24.6	36.1	0.68	1.47
Biersteker 85	uww	Dutch	64.0	47.0	1.36	0.73	11.7	21.5	0.54	1.84
Biersteker 85	uww	Dutch	59.1	42.9	1.38	0.73	12.3	26.6	0.46	2.15
Biersteker 85	uww	Dutch	68	51	1.33	0.73	12.3	23.3	0.53	1.89
Pichard 00	dexa	Swiss	64	47	1.36	0.72	19.3	28.5	0.67	1.48
Steffenson 02	3c	Denmark	66.3	54.1	1.23	0.82	11.8	17.9	0.66	1.51
Steffenson 02	3c	Denmark	66.0	47.2	1.40	0.72	13.4	20.0	0.67	1.49
Steffenson 02	3c	Denmark	69.3	46.7	1.48	0.67	16.4	28.2	0.58	1.72
Kyle 04	bia	Swiss	60.1	43.4	1.38	0.72	20.4	27.2	0.75	1.33
Kyler 05	bia	Swiss	60.0	43.3	1.39	0.72	20.5	27.5	0.75	1.34
Kyler 05	bia	Swiss	60.5	43.2	1.40	0.71	19.3	26.8	0.72	1.39
Kyler 05	bia	Swiss	60.4	43.2	1.40	0.72	17.7	26.7	0.66	1.51
Beddoe 98	neutron	UK	59.4	42.1	1.41	0.71	22.9	27.9	0.82	1.22
Larsson 03	tbk	Sweden	61.9	43.0	1.44	0.69	23.3	35.4	0.66	1.52
Larsson 03	tbk	Sweden	61.3	42.7	1.44	0.70	23.9	35.8	0.67	1.50
Illner 00	dexa	Germany	63.1	45.9	1.37	0.73	12.7	26.9	0.47	2.11
Malavolti 03	dexa	Italy	58.3	41.6	1.40	0.71	22.7	35.6	0.64	1.57
Jaffrin 05	dexa	France	60.7	43.1	1.41	0.71	19.9	31.2	0.64	1.57
Kyle 01a	bia	Swiss	60.3	43.3	1.39	0.72	18.5	28.4	0.65	1.54
Kyle 01a	bia	Swiss	60.2	42.9	1.40	0.71	20.2	26.9	0.75	1.34
Kyle 01a	bia	Swiss	58.5	42.6	1.37	0.73	21.3	27.4	0.78	1.29
Kyle 01a	bia	Swiss	59.1	42.4	1.39	0.72	15.7	27.3	0.58	1.74
Kyle 01b	bia	Swiss	62.8	44.9	1.40	0.71	23.8	33.1	0.72	1.39
Kyle 01b	bia	Swiss	62.6	45	1.39	0.72	23.2	28.7	0.81	1.24
Kyle 01b	bia	Swiss	63.4	45.4	1.40	0.72	19.8	26.5	0.75	1.34
Kyle 01b	bia	Swiss	62.2	45.1	1.38	0.73	17.3	26.9	0.64	1.56
Kyle 01c	bia	Swiss act	61.4	43.9	1.40	0.71	20.1	26.6	0.75	1.33
Kyle 01c	bia	Swiss act	60.7	43.4	1.40	0.71	18.6	26.7	0.70	1.43
Kyle 01c	bia	Swiss sed	61.8	43.9	1.41	0.71	14.8	22.7	0.65	1.54
Kyle 01c	bia	Swiss sed	60.9	43	1.42	0.71	16.5	23.8	0.69	1.44
Te Velde 04	dexa	Dutch	61.7	42.6	1.45	0.69	27.0	37.4	0.72	1.39
Kirchengast 10	bia	Austria					18.2	24.9	0.73	1.37
Kirchengast 10	bia	Austria					12.1	25.6	0.47	2.12
Kirchengast 10	bia	Austria					18.8	22.8	0.82	1.21
Kirchengast 10	bia	Austria					13.3	24.3	0.55	1.83
Clarys84	dissect	Belgium	47.7	42.0	1.14	0.88	28.1	40.5	0.69	1.44
Withers 98	4c	Australia	59.7	48.2	1.24	0.81	12.1	16.2	0.75	1.34
Withers 98	4c	Australia	56.9	39.6	1.44	0.70	22.4	28.5	0.79	1.27
Piers 00	deut	Australia	61.9	44.3	1.40	0.72	19.7	28.7	0.69	1.45
Weber 00	dexa	Australia	58.0	43.0	1.35	0.74	25.5	31.4	0.81	1.23
Janmahazatian 05	dexa	Australia	72.2	50.5	1.43	0.70	33.4	46.0	0.73	1.38
Withers 91	uww	Australia	67.1	48.2	1.39	0.72	10.1	18.5	0.55	1.83
Taylor 10	dexa	Australia	61.9	41.4	1.50	0.67	17.3	33.0	0.52	1.91
George 89	uww he	Canada	59.1	40.2	1.47	0.68	27.7	42.2	0.66	1.52

			fat free mass				% fat			
**reference**	**method**	**pop**	**m**	**f**	**m/f**	**f/m**	**m**	**f**	**m/f**	**f/m**
George 89	uww he	Canada	56.7	41.9	1.35	0.74	18.1	33.9	0.53	1.88
Carter 01	dexa	Canada	65.9	50.3	1.31	0.76	15.6	26.2	0.60	1.68
George 89	uww he	Canada	58.3	38.1	1.53	0.65	12.1	27.2	0.44	2.25
George 89	uww he	Canada	56.2	41.5	1.35	0.74	3.8	16.0	0.24	4.25
Dionne 99	uww	Canada	65.2	47.3	1.38	0.73	26.5	38.0	0.70	1.43
Buchholz 01	tbw	Canada	59.8	41.9	1.43	0.70	19.3	29.5	0.66	1.53
Sun 05	dexa	Canada	63.2	43.3	1.46	0.68	25.0	36.7	0.68	1.47
Tahara 03	dexa	Jap.Am	54.5	38.9	1.40	0.71	8.5	22.9	0.37	2.71
Tahara 02	uww	Japan	53.9	39.7	1.36	0.74	17.5	24.8	0.71	1.42
Tanaka 02	uww	Japan	55.4	40.2	1.38	0.73	12.5	23.4	0.53	1.88
Tsunenari 93	dexa	Japan	51.7	34.5	1.50	0.67	21.7	38.2	0.57	1.76
Tsunenari 93	dexa	Japan	50.0	34.3	1.46	0.69	18.7	34.0	0.55	1.82
Tsunenari 93	dexa	Japan	40.8	31.4	1.30	0.77	26.8	36.6	0.73	1.37
Abe 03	hw	Japan	56.3	40.8	1.38	0.72	11.3	26.6	0.43	2.35
Tahara 03	dexa	Japan	54.5	38.9	1.40	0.71	19.4	30.0	0.65	1.55
Lusaki 90	hd	US	72.6	51.1	1.42	0.70	13.2	21.1	0.62	1.61
Lemmer 01	dexa	US	62.9	42.9	1.47	0.68	25.5	33.7	0.76	1.32
Segal 88	dens	US	66	48	1.38	0.73	16.5	32.4	0.51	1.97
Segal 88	dens	US	67	45	1.49	0.67	23.9	28.6	0.84	1.20
Segal 88	dens	US	62	44	1.41	0.71	21.5	27.9	0.77	1.30
Segal 88	dens	US	61	43	1.42	0.70	18.7	14.0	1.33	0.75
Wang 01	mri	**US**	63.1	43.8	1.44	0.69	21.6	34.9	0.62	1.62
Horlick 00	dexa	**US**	60.3	42.9	1.41	0.71	12.9	32.3	0.40	2.51
Nielson 03	dexa	US	67.4	42.1	1.60	0.62	23.2	35.7	0.65	1.54
Pietrobelli 02	tbw trit	US	53.2	41.5	1.28	0.78	20.6	35.8	0.58	1.74
Paul 04	dexa	US	63.1	45.6	1.38	0.72	24.2	38.5	0.63	1.60
Proctor 99	uww	US	59.8	45.0	1.33	0.75	16.2	21.7	0.75	1.34
Proctor 99	uww	US	59.0	41.8	1.41	0.71	17.4	27.3	0.64	1.57
Sparti 97	4c	US	60.4	44.4	1.36	0.74	20.1	31.4	0.64	1.56
Presta 83	uww	US	63.1	45.2	1.40	0.72	24.6	29.3	0.84	1.19
Castro 95	hw	US	61.5	43.8	1.41	0.71	4.3	13.9	0.31	3.22
Castro 95	hw	US	61.5	43.8	1.41	0.71	24.5	29.0	0.85	1.18
Ferraro 92	hd	US	63.2	47.6	1.33	0.75	24.9	41.2	0.60	1.66
Lindle 97	dexa	US	61.4	42.4	1.45	0.69	29.3	38.6	0.76	1.32
Lindle 97	dexa	US	58.6	39.8	1.47	0.68	29.5	39.5	0.75	1.34
Chumlea 88	uww	US	63.7	43.7	1.46	0.69	23.5	31.9	0.74	1.36
Barlett 91	uww	US	62.5	47.6	1.31	0.76	25.4	35.4	0.72	1.39
Barlett 91	uww	US	64.6	45.6	1.42	0.71	20.7	26.7	0.78	1.29
Barlett 91	uww	US	61.5	47.6	1.29	0.77	17.6	30.7	0.57	1.75
Barlett 91	uww	US	62.0	45.0	1.38	0.73	21.3	27.9	0.76	1.31
Nelson 92	uww	US	66.2	46.1	1.44	0.70	23.4	36.6	0.64	1.56
Lesser 79	gas	US	59.8	45.0	1.33	0.75	18.6	23.8	0.78	1.28
Bossingham 05	uww	US	59.9	44.6	1.34	0.74	23.8	31.3	0.76	1.31
Cohn 85	dexa	US	63.3	45.5	1.39	0.72	24.2	29.9	0.81	1.24
Cohn 85	dexa	US	63.3	42.9	1.48	0.68	78.1	77.6	1.01	0.99
Cohn 85	dexa	US	66.7	45.8	1.46	0.69	7.0	33.5	0.21	4.81
Bishop 87	uww	US	63.0	47.6	1.32	0.76	13.3	22.5	0.59	1.68

			fat free mass				%fat			
**reference**	**method**	**pop**	**m**	**f**	**m/f**	**f/m**	**m**	**f**	**m/f**	**f/m**
Bishop 87	uww	US	60.2	44.9	1.34	0.75	16.6	25.7	0.65	1.54
Ivey 00	dexa	US	62.3	43.0	1.45	0.69	24.3	31.9	0.76	1.31
Luke 02	tbw	US	50.6	39.9	1.27	0.79	23.7	51.1	0.46	2.15
Kim 05	dexa	US	59.5	38.9	1.53	0.65	25.2	38.3	0.66	1.52
Abe 00	dexa	US	62.4	42.3	1.48	0.68	28.5	33.5	0.85	1.17
Abe 00	dexa	US	61.1	41.0	1.49	0.67	30.3	36.6	0.83	1.21
Jozsi 99	bia	US	59.3	43.0	1.38	0.73	21.6	25.0	0.86	1.16
Kyler 05	bia	US	63.6	45.0	1.41	0.71	26.0	36.4	0.72	1.40
Kyler 05	bia	US	64.6	44.8	1.44	0.69	23.1	35.2	0.66	1.52
Kyler 05	bia	US	61.3	42.8	1.43	0.70	22.6	32.3	0.70	1.43
Peterrson 03	4c	US	58.5	42.3	1.38	0.72	25.9	33.4	0.78	1.29
Kohrt 98	uww	US	63.1	44.7	1.41	0.71	22.9	28.9	0.79	1.27
Kohrt 98	uww	US	65.7	45.3	1.45	0.69	14.9	21.9	0.68	1.47
Kriketos 00	uww	US	64.8	44.8	1.45	0.69	24.2	26.6	0.91	1.10
Kriketos 00	uww	US	61.6	42.3	1.46	0.69	14.1	22.7	0.62	1.61
Sun 03	tbw	US	62.9	46.8	1.34	0.74	21.3	36.3	0.59	1.71
Sun 03	tbw	US	58.8	43.4	1.35	0.74	22.2	33.6	0.66	1.51
Sun 03	tbw	US	58.0	41.6	1.39	0.72	23.2	35.1	0.66	1.51
Wetter 04	bia	US	61.7	44.8	1.38	0.73	16.6	26.3	0.63	1.58
Gallagher 05	mri	US As	61.4	46.1	1.33	0.75	22.9	33.3	0.69	1.46
Evans 01	dexa	US	74.7	45.9	1.63	0.61	20.1	35.7	0.56	1.78
Gallagher 05	mri	US B	61.4	46.1	1.33	0.75	23.3	40.7	0.57	1.74
Ruby 02	hw	US	62.3	51.2	1.22	0.82	8.8	14.5	0.61	1.65
Chumlea 02	bia	US hisp	59.8	43.5	1.37	0.73	27.1	40.7	0.66	1.50
Chumlea 02	bia	US hisp	60.2	42.6	1.41	0.71	27.1	40.3	0.67	1.48
Chumlea 02	bia	US hisp	58.1	42.8	1.36	0.74	25.9	39.4	0.66	1.52
Chumlea 02	bia	US hisp	61.3	42.8	1.43	0.70	22.6	32.3	0.70	1.43
Falkel 85	hw	US Ma	66.8	46.1	1.45	0.69	13.1	21.3	0.62	1.62
Toth 94	uww	US Md	68.0	46.0	1.48	0.68	16.0	25.8	0.62	1.61
Poehlman 95	uww	US Md	64.1	45.1	1.42	0.70	17.5	27.5	0.64	1.57
Chumlea 02	bia	US nhb	62.2	48.2	1.29	0.77	25.6	40.9	0.63	1.60
Chumlea 02	bia	US nhb	61.9	47.3	1.31	0.76	26.0	41.4	0.63	1.59
Chumlea 02	bia	US nhb	64.6	45.4	1.42	0.70	24.5	39.5	0.62	1.61
Chumlea 02	bia	US nhb	62.6	46.4	1.35	0.74	25.0	37.1	0.67	1.48
Chumlea 02	bia	US nhw	64.6	44.8	1.44	0.69	25.7	38.6	0.67	1.50
Chumlea 02	bia	US nhw	63.6	45	1.41	0.71	24.9	36.6	0.68	1.47
Chumlea 02	bia	US nhw	62.2	44.3	1.40	0.71	24.3	34.9	0.70	1.44
Chumlea 02	bia	US nhw	59.8	43.5	1.37	0.73	22.6	32.3	0.70	1.43
Gallagher 96	tbk	US	60.2	42.6	1.41	0.71	20.6	41.1	0.50	2.00
Gallagher 98	dexa	US	58.1	42.8	1.36	0.74	15.8	21.7	0.73	1.38
Visser 97	4c	US	61.3	42.8	1.43	0.70	22.1	36.2	0.61	1.64
Visser 97	4c	US	60.4	42.3	1.43	0.70	22.1	31.4	0.70	1.43
Welle 90	tbk	US	63.6	51.3	1.24	0.81	13.8	28.3	0.49	2.04
Cohn 83	tbk	US	57.1	42.0	1.36	0.74	26.4	34.0	0.78	1.29
Wellens 94	dexa	US	58.9	40.4	1.46	0.69	21.7	35.7	0.61	1.65
Guo 97	uww	US	60.2	44.0	1.37	0.73	13.9	27.0	0.51	1.94
Guo 97	uww	US	57.4	43.7	1.32	0.76	12.2	25.8	0.47	2.11

reference	method	pop	fat free mass m	f	m/f	f/m	%fat m	f	m/f	f/m
Demerath 99	4c	US	59.8	44.5	1.34	0.74	16.6	26.2	0.63	1.58
Gallagher 05	mri	US w	61.4	46.1	1.33	0.75	25.3	36.9	0.69	1.46
Nindl 02	dexa	US	57.8	40.0	1.45	0.69	30.7	39.7	0.77	1.29
NHANES 99-06	dexa	US 16-49	56.4	39.7	1.42	0.70	23.1	35.0	0.77	1.52
Weyer 99	uww/dexa	US/Pima	63.5	49.0	1.30	0.77	30.6	44.9	0.68	1.47
Biaggi 99	uww	US	67.1	47.9	1.40	0.71	21.5	28.6	0.75	1.33
Biaggi 99	bia	US	67.1	49.4	1.36	0.74	20.2	27.5	0.73	1.36
Wang 01	mri	US	63.1	43.8	1.44	0.69	21.6	34.9	0.62	1.62
Zilhman 15	dissect	US	49.7	36.5	1.36	0.73	20.3	36.3	0.56	1.79
Lassek 09	dexa	US	60.8	43.1	1.41	0.71	23.2	33.9	0.68	1.46
Gallagher 97	ct scan	US	61.4	44.9	1.37	0.73	21.8	34.7	0.63	1.59

bia=bioelectrical impedance; deut=deuterium; dexa=dual energy x-ray aborptiometry
3c, 4c=3 or 4 compartment; tbk=total body potassium; uww= underwater weighing

Table S2. Sex differences in muscle mass

reference	country	method	male	sd	female	sd	ratio	*d*
**total muscle**								
average							1.65	2.70
NHANES 99-06*	US 15-50	dexa	25.0	4.0	16.4	2.7	1.52	2.71
Sousa 03	Brazil	tbk	105	11	73	10	1.44	3.05
Sousa 03	Brazil	tbk	142	18	84	11	1.69	4.00
Glenmark 94	Sweden	biopsy	55	12	51	9	1.08	0.38
Glenmark 94	Sweden	biopsy	55	12	48	13	1.15	0.56
Proctor 99	US	creatinine	34.9	5.8	26.3	4.4	1.33	1.69
Phillips 95	US	creatinine	1642	418	1041	291	1.58	
Cunngham 82	US	creatinine	28		17		1.65	
Philllips 95	UK	creatinine	26.3	7.6	15.4	5.9	1.71	1.61
Frontera 91	US	creatinine	27.9		18.3		1.52	
Frontera 91	US	creatinine	26.6		16.8		1.58	
Heymsfield 02	US	CT scan	30		17		1.76	
Illner 00	Germany	dexa	28.8		17.9		1.61	
Illner 00	Germany	dexa	28.8	3	17.9	2.5	1.61	3.96
Neder 99	Brazil	dexa	18.9	1.2	14.9	1.6	1.27	2.86
Hays 02	US	dexa	33.3	5	19.7	3.1	1.69	3.36
Janssen 00	Canada US	mri	33		21		1.57	
Wang 01	US	mri	33.4		20.7		1.61	
Gallagher 98	US	mri	35.7	3.8	18.9	3.2	1.89	4.80
Kim 04	US	mri	31.7	5.9	19.8	3.9	1.60	2.43
Shen 04	US	mri	31.2	5.4	20.5	4.1	1.52	2.25
Abe 03	Japan	mri	22.3	3	13.5	2	1.65	3.52
Lan 89	China	tbk	124.02		86.57		1.43	
Wormersly 76	Scots muscular	tbk	4760	660	3290	381	1.45	2.82
Wormersly 76	Scots sed	tbk	3790	486	2500	278	1.52	3.38
Lahham 98	Slovak	tbk	143	15	110	11	1.30	2.54
Wang 03	US	tbk	4110	567	2405	324	1.71	3.83
Wang 03	US	tbk	34.3	5.8	19.9	3.3	1.72	3.16
Ellis 75	US	tbk	136.4	25.2	84.7	10.3	1.61	2.91
Heymsfield 90	US	tbk	17.1	7.1	5	4.6	3.42	2.07
Heymsfield 90	US	tbk	21.4	8	7	5.9	3.06	2.07
Cohn 83	US	tbk	21.1	25	11	32	1.92	0.35
He 03	US	tbk	4146		2617		1.58	
He 03	US	tbk	3835		2422		1.58	
He 03	US	tbk	4065		2563		1.59	
He 03	US	tbk	3969		2442		1.63	
Gallagher 97	US b	tbk/tbn	29.4	4.4	20.5	2.8	1.43	2.47
Gallagher 97	US w	tbk/tbn	28.33	3.9	18.6	2.6	1.52	2.99
Wetter 04	US	ultrasound	33.5	3.1	21.8	2.1	1.54	4.50
Zihlman 15	US	dissection	39.4		28.6		1.38	

* new analysis, tbk = total body potassium; tbn = total body nitrogen
;

Table S3 Sex differences in arm and leg muscle

source		m	f	ratio	d
**arm muscle**				1.72	2.91
NHANES*	US	7.0	4.0	1.52	3.01
Abe 03	Japan	2.3	1.2	1.92	4.40
Janssen 02	UK	13.9	8.2	1.70	
Nindl 02	US	8.7	4.9	1.78	
Gallagher 97	US b	7.7	4.9	1.57	2.24
Gallagher 97	US w	7.2	4.1	1.76	2.48
Fuller 92	UK	4.8	2.8	1.71	
Lassek 09	US	2.5	1.4	1.79	2.44
**leg muscle**				1.48	2.37
NHANES*	US	25.0	16.4	1.52	2.71
Janssen 00	US	17.2	11.6	1.48	
Gallagher 97	US b	21.7	15.6	1.39	2.26
Gallagher 97	US w	21.1	14.5	1.46	2.81
Fuller 92	UK	17.4	11.4	1.53	
Fullller 99	UK	7.4	4.0	1.85	2.83
Lawler 98	US	20.3	13.7	1.48	2.24
Lawler 98	US	22.3	14.9	1.50	3.22
Shih 00	US	14.4	9.8	1.47	2.14
Shih 00	US	7.8	5.2	1.50	1.86
Abe 03	Japan	2.4	1.8	1.33	2.00
Abe 03	Japan	8.0	5.0	1.60	3.53
Nindl 02	US	20.9	14.8	1.41	0.00
Neder 99	Brazil	18.9	14.9	1.27	2.86

* new analysis

REFERENCES FOR SUPPLEMENTARY TABLES

Abe, T., Dehoyos, D.V., Pollock, M.L., and Garzarella, L. (2000). Time course for strength and muscle thickness changes following upper and lower body resistance training in men and women. *Eur J Appl Physiol* 81**,** 174-180.

Abe, T., Kearns, C.F., and Fukunaga, T. (2003). Sex differences in whole body skeletal muscle mass measured by magnetic resonance imaging and its distribution in young Japanese adults. *Br J Sports Med* 37**,** 436-440.

Bartlett, H.L., Puhl, S.N., Hodgson, J.L., and Buskirk, E.R. (1991). Fat-free mass in relation to stature: ratios of fat-free mass to height in children, adults, and elderly subjects. *Am J Clin Nutr* 53**,** 1112-1116.

Beddoe, A.H., and Samat, S.B. (1998). Body fat prediction from skinfold anthropometry referenced to a new gold standard: in vivo neutron activation analysis and tritium dilution. *Physiol Meas* 19**,** 393-403.

Biaggi, R.R., Vollman, M.W., Niles, M.A., Brener, C.E., and Flakoll, P.F. (1999). Comparison of air-displacement plethysmography with hydrostatic weighing and bioelectrical impedance analysis for the assessment of body composition in healthy adults. *Am J Clin Nutr* 69**,** 898-903.

Biersteker, M.W.A., and A., P. (1985). Vital capacity in trained and untrained healthy young adults in the Netherlands. *Eur J Appl Physiol* 54**,** 46-53.

Bishop, P., Cureton, K., and Collins, M. (1987). Sex difference in muscular strength in equally-trained men and women. *Ergonomics* 30**,** 675-687.

Boot, A.M., Bouquet, J., Ridder, M.a.J.D., Krenning, E.P., and Keizer-Shrama, S.M. (1997). Determinants of body composition measured by dual-energy x-ray absorptiometry in Dutch children and adolescents. *Am J Clin Nutr* 66**,** 232-238.

Bossingham, M.J., Carnell, N.S., and Campbell, W.W. (2005). Water balance, hydration status, and fat-free mass hydration in younger and older adults. *Am J Clin Nutr* 81**,** 1342-1350.

Brodie, D.A., and Eston, R.G. (1992). Body fat estimations by electrical impedance and infra-red interactance. *Int J Sports Med* 13**,** 319-325.

Buchholz, A.C., Rafii, M., and Pencharz, P.B. (2001). Is resting metabolic rate different between men and women? *Brit J Nutrition* 86**,** 641-646.

Carter, S.L., Rennie, C.D., Hamilton, S.J., and Parnopolsky, M.A. (2001). Changes in sketal muscle in males and females following endurance training. *Can J Physiol Pharm* 79**,** 386-392.

Castro, M.J., Mccann, D.J., Shaffrath, J.D., and Adams, W.C. (1995). Peak torque per unit cross-sectional area differs between strength-trained and untrained young adults. *Med & sci in sp & ex* 27**,** 397-403.

Chumlea, W.C., Baumgartner, R.N., and Roche, A.F. (1988). Specific resistivity used to estimate fat-free mass from segmental body measures of bioelectrical impedance. *Am J Clin Nutr* 48**,** 7-15.

Chumlea, W.C., Guo, S.S., Kuczmarski, R.J., Flegal, K.M., Johnson, C.L., Heymsfield, S.B., Lukaski, H.C., Friedl, K., and Hubbard, V.S. (2002). Body composition estimates from NHANES III bioelectrical impedance data. *Int J Obesity* 26**,** 1596-1609.

Clarys, J.P., Martin, A.D., and Drinkwater, D.T. (1984). Gross tissue weights in the human body by cadaver dissection. *Human biology* 56**,** 459-473.

Cohn, S.H., Vartsky, D., Yasumra, S., Vaswani, A.N., and Ellis, K.J. (1983). Indexes of body cell mass: nitrogen versus potassium. *Am J Physiology* 244**,** E305-E310.

Cohn, S.H., Vaswani, A.N., Yasumura, S., Yuen, K., and Ellis, K.J. (1985). Assessment of cellular mass and lean body mass by noninvasive nuclear techniques. *J Lab Clin Med* 105**,** 305-311.

Cunningham, J.J. (1982). Body composition and resting metabolic rate: the myth of feminine metabolism. *Am J Clin Nutr* 36**,** 721-726.

Demerath, E.W., Towne, B., Wisemandle, W., Blangero, J., Chumlea, W.C., and Siervogel, R.M. (1999). Serum leptin concentration, body composition, and gonadal hormones during puberty. *Int J Obesity* 23**,** 678-685.

Dionne, I., Despres, J.P., Bouchard, C., and Tremblay, A. (1999). Gender difference in the effect of body composition on energy metabolism. *Int J Obesity* 23**,** 312-319.

Ellis, K.J., and Cohn, S.H. (1975). Correlation between skeletal calcium mass and muscle mass in man. *J Appl*

Physiol 38, 455-460.
Evans, E.M., Prior, B.M., Arngrimsson, S.A., Modlesky, C.M., and Cureton, K.J. (2001). Relation of bone mineral density and content to mineral content and density of the fat-free mass. *J Appl Physiol* 91, 2166-2172.
Falkel, J.E., Sawka, M.N., Levine, L., and Pandolf, K.B. (1985). Upper to lower body muscular strength and endurance ratios for women and men. *Ergonomics* 28, 1661-1670.
Ferraro, R., Lilloja, S., Fontvieille, A.M., Rising, R., Bogardus, C., and Ravussin, E. (1992). Lower sedentary metabolic rate in women compared with men. *J Clin Invest* 90, 780-784.
Frontera, W.R., Hughes, V.A., Lutz, K.J., and Evans, W.J. (1991). A cross-sectional study of muscle strength and mass in 45- to 78-yr-old men and women. *J Appl Physiol* 71, 644-650.
Gallagher, D., Belmonte, D., Deurengerg, P., and Wang, Z. (1998). Organ-tissue mass measurement allows modeling of REE and metabolically active tissue mass. *Am J Physiology* 275, E249-E258.
Gallagher, D., Kuznia, P., Heshka, S., Albu, J., Heymsfield, S.B., and Et Al. (2005). Adipose tissue in muscle: a novel depot similar in size to visceral. *Am j clin nut* 81, 903-910.
Gallagher, D., Visser, M., Meersman, R.E.D., and Sepulveda, D. (1997). Appendicular skeletal muscle mass: effects of age, gender, and ethnicity. *J Appl Physiol* 83, 229-239.
Gallagher, D., Visser, M., Wang, Z., Harris, T., Jr, R.N.P., and Heymsfield, S.B. (1996). Metabolically active components of fat-free body mass: influences of age, adiposity, and gender. *Metabolism* 45, 992-997.
Garcia, A.L., Wagner, K., Hothorn, T., Koebnick, C., Zunft, H.F., and Trippo, U. (2005). Improved prediction of body fat by measuring skinfold thickness, circumferences, and bone breadths. *Obesity Research* 13, 626-634.
George, V., Tremblay, A., Despres, J.F., Leblanc, C., Perusse, L., and Bouchard, C. (1989). Evidence for the existence of small eaters and large eaters of similar fat-free mass and activity level. *Int J Obesity* 13, 43-53.
Glenmark, B. (1994). Skeletal muscle fibre types, physical performance, physical activity and attitude to physical activity in women and men, a follow-up from age 16 to 27. *Acta Physiol* 151 s 623, 1-47.
Guo, S.S., Chumlea, W.C., Roche, A.F., and Siervogel, R.M. (1997). Age- and maturity-related changes in body composition during adolescence into adulthood: the Fels Longitudinal Study. *Int J Obesity* 21, 1167-1175.
Han, T.S., Carter, R., Currall, J.E.P., and Lean, M.E.J. (1996). The influence of fat free mass on prediction of denisometric body composition by bioelectrical impedance analysis and by anthropometry. *Eur J Clin Nutr* 58, 542-548.
Hayes, M., Chustek, M., Wang, Z., Gallagher, D., Heshka, S., Spungen, A., Bauman, W., and Heymsfield, S.B. (2002). DEXA: Potential for Creating a Metabolic Map of Organ-Tissue Resting Energy Expenditure Components. *Obesity Research* 10, 969-977.
He, Q., Heo, M., Heshka, S., Wang, J., and N., R.N.P.R. (2003). Total body potassium differs by sex and race across the adult life span. *Am J Clin Nutr* 78, 72-77.
Heitmann, B.L. (1991). Body fat in the adult Danish population aged 35-65 years. *Int J Obesity* 15, 535-545.
Heymsfield, S.B., Mcmanus, C., and Mcmanus, E. (1982). Anthropometric measurement of muscle mass: revised equations for calculating bone-free arm muscle area. *Am J Clin Nutr* 36, 680-690.
Heymsfield, S.B., Smith, R., Aulet, M., Bensen, B., Lichtman, S., Wang, J., and Jr, R.N.P. (1990). Appendicular skeletal muscle mass: measurement by dual-photon absorptiometry. *Am J Clin Nutr* 52, 214-218.
Illner, K., Brinkmann, G., Heller, M., Bosy-Westphal, A., and Müller, M. (2000). Metabolically active components of fat free mass and resting energy expenditure in nonobese adults. *Am J Physiology* 278, E308-E315.
Ivey, F.M., Tracy, B.L., Lemmer, J.T., Ness-Avier, M., and Metter, M.J. (2000). Effects of strength training and detraining on muscle quality: age and gender comparisons. *J Gerontology* 55A, B152-B157.
Jaffrin, M.Y., Kieffer, R., and Moreno, M.V. (2005). Evaluation of a foot-to-foot impedance meter measuring extracellular fluid volume in addition to fat-free mass and fat tissue mass. *Nutrition* 21, 815-824.
Janmahasatian, S., Duffull, S.B., Ash, S., Ward, L.C., Byrne, N.M., and Green, B. (2005). Quantification of lean bodyweight. *Clin Pharmacokinet* 44, 1051-1065.

Janmahazatian, S., Duffull, S.B., Ash, S., Ward, L.C., Byrne, N.M., and Green, B. (2005). Quantification of lean bodyweight. *Clin Pharmacokinet* 44**,** 1051-1065.

Janssen, I., Heymsfield, S.B., Wang, Z., and Ross, R. (2000). Skeletal muscle mass and distribution in 468 men and women aged 18-88 yr. *J Appl Physiol* 89**,** 81-88.

Jozsi, A.C., Campbell, W.W., Joseph, L., Davey, S.L., and Evans, W.J. (1999). Changes in power with resistance training in older and younger men and women. *J Gerontology* 54A**,** M591-M596.

Kanade, A.N., Gokhale, M.K., and Rao, S. (2001). Energy costs of standard activities among Indian adults. *Eur J Clin Nutr* 55**,** 708-713.

Kanehisa, H., Miyatani, M., Azuma, K., Kuno, S., and Fukunaga, T. (2004). Influences of age and sex on abdominal muscle and subcutaneous fat thickness. *Eur J Appl Physiol* 91**,** 534-537.

Kim, J., Heshka, S., Gallagher, D., Kotler, D.P., Mayer, L., Albu, J., Shen, W., Freda, P.U., and Heymsfield, S.B. (2004). Intermuscular adipose tissue-free skeletal muscle mass: estimation by dual-energy X-ray absorptiometry in adults. *J Appl Physiol* 97**,** 655-660.

Kim, J.-S., Cross, J.M., and Bamman, M.M. (2005). Impact of resistance loading on myostatin expression and cell cycle regulation in young and older men and women. *Am J Physiology* 288**,** E1110-E1119.

Kirchengast, S. (2010). Gender differences in body composition from childhood to old age: an evolutionary point of view. *J Life Sci* 2**,** 1-10.

Kohrt, W.M. (1998). Preliminary evidence that DEXA provides an accurate assessment of body composition. *J Appl Physiol* 84**,** 372-377.

Kuriyan, R., Petracchi, C., Ferro-Luzzi, A., Shetty, P.S., and Kurpad, A.V. (1998). Validation of expedient methods for measuring body composition in Indian adults. *Indian J Med Res* 107**,** 37-45.

Kyle, U. (2001). Physical activity and fat-free and fat mass by bioelectrical impedance in 3853 adults. *Med Sci Sports Exerc* 33**,** 576-584.

Kyle, U.G., Genton, L., Karsegard, L., Slosman, D.O., and Pichard, C. (2001). Single Prediction Equation for Bioelectrical Impedance Analysis in Adults Aged 20-94 Years. *Nutrition* 17**,** 248-253.

Kyle, U.G., Genton, L., Lukaski, H.C., Dupertius, Y.M., and Et Al. (2005). Comparison of fat-free mass and body fat in Swiss and American adults. *Nutrition* 21**,** 161-169.

Kyle, U.G., Genton, L., Slosman, D.O., and Pichard, C. (2001). Fat-Free and Fat Mass Percentiles in 5225 Healthy Subjects Aged 15 to 98 Years. *Nutrition* 17**,** 534-541.

Kyle, U.G., Morabia, A., Schutz, Y., and Pichard, C. (2004). Sedentarism affects body fat mass index and fat-free mass index in adults aged 18 to 98 years. *Nutrition* 20**,** 255-260.

Lahham, A., Fulop, M., Vladar, M., and Ragan, P. (1998). Body potassium content and radiation dose from 40K to the Slovak population. *Health Physics* 74**,** 346-349.

Lan, C.-Y., and Weng, P.S. (1989). Body K and 40K in Chinese subjects measured with a whole-body counter. *Health Physics* 57**,** 743-746.

Larsson, I., Lindroos, A.K., Pletonen, M., and Sjostrom, L. (2003). Potassium per kilogram fat-free mass and total body potassium: predictions from sex, age, and anthropometry. *Am J Physiology* 284**,** E416-E423.

Lemmer, J.T., Ivey, F.M., Ryan, A.S., Martel, G.F., Hurlbut, D.E., Metter, J.E., Fozard, J.L., Fleg, J.L., and Hurley, B.F. (2001). Effect of strength training on resting metabolic rate and physical activity: age and gender comparisons. *Med & Sci in* 33**,** 532-541.

Lesser, G.T., and Markofsky, J. (1979). Body water compartments with human aging using fat-free mass as the reference standard. *Am J Physiology* 236**,** R215-R220.

Lindle, R.S., Metter, E.J., Lynch, N.A., Fleg, J.F., and Fozard, J.L. (1997). Age and gender comparisons of muscle strength in 654 women and men aged 20-93 yr. *J Appl Physiol* 83**,** 1581-1587.

Liu, H.Y., Lu, Y.F., and Chen, W.J. (1995). Predictive equations for basal metabolic rate in Chinese adults: a cross-validation study. *Jada* 95**,** 1403-1408.

Lukaski, H.C., Bolonchuk, W.W., Siders, W.A., and Hall, C.B. (1990). Body composition assessment of athletes using bioelectrical impedance measurements. *J Sp Med Phys* 30**,** 434-440.

Luke, A. (2002). Activity energy expenditure and adiposity among black adults in Nigeria and the United States. *Am J Clin Nutr* 75**,** 1045-1050.

Macmillan, M.G., Reid, C.M., Shirling, D., and Passmore, R. (1965). Body composition, resting oxygen consumption, and urinary creatinine in Edinburgh students. *Lancet* 1**,** 728-729.

Malavolti, M., Mussi, C., Poli, M., Famtuzzi, A.L., and Salvioli, G. (2003). Cross-calibration of eight-polar bioelectrical impedance analysis versus. *Ann Hum Biol* 30**,** 380-391.
Neder, J.A., Lerario, M.C., Castro, M.L., Sachs, A., and Nery, L.E. (2001). Peak VO2 correction for fat-free mass estimated by anthropometry and DEXA. *Med & Sci in* 33**,** 1968-1975.
Neder, J.A., Nery, L.E., Silva, A.C., Andreoni, S., and Whipp, B.J. (1999). Maximal aerobic power and leg muscle mass and strength related to age in non-athletic males and females. *Eur J Appl Physiol* 79**,** 522-530.
Nelson, K.M., Weinsier, R.L., Long, C.L., and Schutz, Y. (1992). Prediction of resting energy expenditure from fat-free mass and fat mass. *Am J Clin Nutr* 56**,** 848-856.
Nielsen, S., Guo, Z., Albu, J.B., Klein, S., and Brien, M.D.J. (2003). Energy expenditure, sex, and endogenous fuel availability in humans. *J Clin Invest* 111**,** 981-988.
Nindl, B.C., Scoville, C.R., Sheehan, K.M., Leone, C.D., and Mello, R.P. (2002). Gender differences in regional body composition and somatotrophic influences of IGF-I and leptin. *J Appl Physiol* 92**,** 1611-1618.
Norgan, N.G., Ferro-Luzzi, A., and Durnin, J. (1982). The body composition of New Guinean adults in contrasting environments. *Am J Human Biol* 9**,** 343-353.
Paul, D.R., Novotny, J.A., and Rumpler, W.V. (2004). Effects of the interaction of sex and food intake on the relation between energy expenditure and body composition. *Am J Clin Nutr* 79**,** 385-389.
Peterson, M.J., Czerwinski, S.A., and Siervogel, R.M. (2003). Development and validation of skinfold-thickness prediction equations with a 4-compartment model. *Am J Clin Nutr* 77**,** 1186-1191.
Phillips, D.I.W. (1995). Relation of fetal growth to adult muscle mass and glucose tolerance. *Diabetic Med* 12**,** 686-690.
Pichard, C., Kyle, U.G., Bracco, D., Slosman, D.O., Morabia, A., and Schutz, T. (2000). Reference values of fat-free and fat masses by bioelectrical impedance analysis in 3393 healthy subjects. *Nutrition* 16**,** 245-254.
Piers, L.S., Soares, M.J., Frandsen, S.L., and O'dea, K. (2000). Indirect estimates of body composition are useful for groups but unreliable in individuals. *Int J Obesity* 24**,** 1145-1152.
Pietrobelli, A., Allison, D.B., Heshka, S., Heo, H., Wang, Z.M., and Bertkau, A. (2002). Sexual dimorphism in the energy content of weight change. *Int J Obesity* 26**,** 1339-1348.
Poehlman, E.T., and Toth, M.J. (1995). Mathematical ratios lead to spurious conclusions regarding age- and sex-related differences in resting metabolic rate. *Am J Clin Nutr* 61**,** 482-485.
Pongchaiyakul, and Chatlert (2005). Prediction of Percentage Body Fat in Rural Thai Population Using Simple Anthropometric Measurements. *Obesity Research* 13**,** 729-738.
Presta, E., Segal, K.R., Gutin, B., Harrison, G.G., and Italie, T.B.V. (1983). Comparison in man of total body electrical conductivity and lean body mass derived from body density: validation of a new body composition method. *Metabolism* 32**,** 524-527.
Proctor, D.N., O'brien, P.C., Atkinson, E.J., and Nair, K.S. (1999). Comparison of techniques to estimate total body skeletal muscle mass in people of different age groups. *Am J Physiology* 40**,** E489-E495.
Rico, H., Revilla, M., Hernandez, E.R., Villa, L.F., and Buergo, M.a.D. (1992). Sex differences in the acquisition of total bone mineral mass peak assessed through dual-energy x-ray absorbtiometry. *Calc Tissue Int* 51**,** 251-254.
Rico, H., Revilla, M., Villa, L.F., Hernandez, E.R., Buergo, M.a.D., and Villa, M. (1993). Body composition in children and Tanner's stages: a study with dual-energy x-ray absorptiometry. *Metabolism* 42**,** 967-970.
Ruby, B.C., Coogan, A.R., and Zderic, T.W. (2002). Gender differences in glucose kinetics and substrate oxidation during exercise near the lactate threshold. *J Appl Physiol* 92**,** 1125-1132.
Schutz, Y., Kaye, U.U.G., and Pichard, C. (2002). Fat-free mass index and fat mass index percentiles in Caucasians aged 18-98 y. *International Journal of Obesity* 26**,** 953-960.
Segal, K.R., Loan, M.V., Fitzgerald, P.J., Hodgdon, J.A., and Itallie, T.B.V. (1988). Lean body mass estimation by bioelectrical impedance analysis: a four-site cross-validation study. *Am J Clin Nutr* 47**,** 7-14.
Shen, W., Punyanitya, M., Wang, Z.M., Gallagher, D., and St. Onge, M.P. (2004). Total body skeletal muscle and adipose tissue volumes: estimation from a single abdominal cross-sectional image. *J Appl Physiol* 97**,** 2333-2338.

Snodgrass, J.J. (2005). Basal Metabolic Rate in the Yakut (Sakha) of Siberia. *Am J Human Biol* 17**,** 155-172.
Sousa, W., Dantos, B.M., and Lipsztein, J.L. (2003). In vivo measurement technique of 40K as an indicator of the amount of skeletal muscle tissue in the body. *Rad Protect Dens* 105**,** 487-490.
Sparti, A., Delany, J.P., Bretonne, J.a.D.J.G.E., and Bray, G.A. (1997). Relationship between resting metabolic rate and the composition of the fat-free mass. *Metabolism* 46**,** 1225-1230.
Steffensen, C.H., Roepstorff, C., Madsen, M., and Kiens, B. (2002). Myocellular triacylglycerol breakdown in females but not in males dring exercise. *Am J Physiology* 282**,** E634-E642.
Stewart, S.P., Bramley, P.N., Heighton, R., Green, J.H., Horsman, A., Losowsky, M.S., and Smith, M.A. (1993). Estimation of body composition from bioelectircal impedance of body segments: comparison with dual-energy x-ray absorptiometry. *Brit J Nutrition* 69**,** 645-666.
Sun, G., French, C.R., Martin, G.R., Younghusband, B., and Etal, R.C.G. (2005). Comparison of multifrequency bioelectrical impedance analysis with dual-energy X-ray absorptiometry for assessment of percentage body fat in a large, healthy population. *Am J Clin Nutr* 81**,** 74-78.
Sun, S.S., Chumlea, C., Heymsfield, S.N., Lukaski, H.C., and Lukaski (2003). Development of bioelectrical impedance analysis prediction equations for body composition with the use of a multicomponent model for use in epidemiologic surveys. *Am J Clin Nutr* 77**,** 331-340.
Tahara, Y., Moji, K., Muraki, S., Honda, S., and Aoyagi, K. (2003). Comparison of body size and composition between young adult Japanese-Americans and Japanese nationals in the 1980s. *Ann Hum Biol* 30**,** 392-401.
Tahara, Y., Moji, M., Aoyagi, K., Tsunawake, N., Mmuraka, S., and Mascie-Taylor, C.G.N. (2002). Age-related pattern of body density and body composition of Japanese men and women 18-59 years of age. *Am J Human Biol* 14**,** 743-752.
Tanaka, S., Itoh, Y., and Hattori, K. (2002). Relationship of body composition to body-fatness estimation in Japanese University students. *Obesity Research* 10**,** 590-596.
Taylor, R.W., Grant, A.M., Williams, S.M., and Goulding, A. (2010). Sex differences in regional body fat distribution from pre-to postpuberty. *Obesity* 18**,** 1410-1416.
Te Velde, S., Twisk, J.W.R., Mechelen, W.V., and Kemper, H.C.G. (2004). Birth weight and musculoskeletal health in 36-year-old men and women: Results from the Amsterdam Growth and Health Longitudinal Study. *Osteoporosis Int* 15**,** 382-388.
Toth, M.J., Gardner, A.W., Ades, P.A., and Poehlman, E.T. (1994). Contribution of body composition and physical activity to age-related decline in peak VO2 in men and women. *J Appl Physiol* 77**,** 647-652.
Tsunenari, T., Tsutsumi, M., Ohno, K., Yamamoto, Y., Kawakatsu, M., and Et Al. (1993). Age- and gender-related changes in body composition in Japanese subjects. *J Bone Min Res* 8**,** 397-402.
Visser, M., Gallagher, D., Deurenberg, P., Wang, J., Jr, R.N.P., and Heymsfield, S.B. (1997). Density of fat-free body mass: relationship with race, age, and level of fatness. *Am J Physiology* 272**,** E781-E787.
Wang, J., and Deurenberg, P. (1996). The validity of predicted body composition in Chinese adults from anthropometry and bioelectrical impedance in comparison with densitometry. *Brit J Nutrition* 76**,** 175-182.
Wang, Z., Heo, M., Lee, R.C., Kotler, D.P., Withers, R.T., and Heymsfield, S.B. (2001). Muscularity in adult humans: proportion of adipose tissue-free body mass as skeletal muscle. *Am J Human Biol* 13**,** 612-619.
Wang, Z., Heo, M., Lee, R.C., Kotler, D.P., Withers, R.T., and Heymsfield, S.B. (2001). Muscularity in adult humans: Proportion of adipose tissue-free body mass as skeletal muscle. *Am J Human Biol* 13**,** 612-619.
Wang, Z., Zhu, S., Wang, J., Jr, R.N.P., and Heyms=Field, S.B. (2003). Whole-body skeletal muscle mass: development and validation of total-body potassium prediction models. *Am J Clin Nutr* 77**,** 76-82.
Weber, C.L., and Schneider, D.A. (2000). Maximal accumulated oxygen deficit expressed relative to the active muscle mass for cycling in untrained male and female subjects. *Eur J Appl Physiol* 82**,** 255-261.
Welle, S., and Nair, K.S. (1990). Relationship of resting metabolic rate to body composition and protein turnover. *Am J Physiology* 258**,** E990-E998.
Wellens, R., Chumlea, W.C., Guo, S., Roche, A.F., and Reo, N.V. (1994). Body composition in white adults by dual-energy x-ray absorptiometry, densitometry, and total body water. *Am J Clin Nutr* 59**,** 547-555.
Werkman, A., Deurenberg-Yap, M., Schmidt, G., and Deurenberg, P. (2000). A comparison between composition and density of the fat-free mass of young adult Singaporean Chinese and Dutch Caucasians.

Ann Nutr & Metab 44**,** 235-242.
Wetter, A.C., and Economos, C.D. (2004). Relationship between quantitative ultrasound, anthropometry and sports participation in college aged adults. *Osteoporosis Int* 15**,** 799-806.
Weyer, C., Snitker, S., Rising, R., Bogardus, C., and Ravussin, E. (1999). Determinants of energy expenditure and fuel utilization in man: effects of body composition, sex, ethnicity and glucose tolerance in 916 subjects. *Int J Obesity* 23**,** 715-722.
Withers, R.T., Craig, N.P., Ball, C.T., Norton, K.I., and Whittingham, N.O. (1991). The Drinkwater-Ross anthropometric fractionation of body mass: comparison with measured body mass and densitometrically estimated fat and fat-free masses. *J Sports Sci* 9**,** 299-311.
Withers, R.T., Laforgia, J., Pillans, R.K., Schipp, N.J., and Chatterton, B.E. (1998). Comparisons of two-, three-, and four-compartment models of body composition analysis in men and women. *J Appl Physiol* 85**,** 238-245.
Womersley, J., Durnin, J., Boody, K., and Mahaffy, M. (1976). Influence of muscular development, obesity, and age on the fat-free mass of adults. *J Appl Physiol* 41**,** 223-229.
Zihlman, A.L., and Bolter, D.R. (2015). Body composition in Pan paniscus compared with Homo sapiens has implications for changes during human evolution. *Proc Natl Acad Sci U S A* 112**,** 7466-7471.
